# Mobility assessment of a rural population in the Netherlands using GPS measurements

**DOI:** 10.1186/s12942-017-0103-y

**Published:** 2017-08-09

**Authors:** Gijs Klous, Lidwien A. M. Smit, Floor Borlée, Roel A. Coutinho, Mirjam E. E. Kretzschmar, Dick J. J. Heederik, Anke Huss

**Affiliations:** 1 Julius Centre for Health Sciences and Primary Care, University Medical Centre Utrecht, Utrecht, The Netherlands; 2 Institute for Risk Assessment Sciences (IRAS), Division Environmental Epidemiology and Veterinary Public Health (EEPI-VPH), Utrecht University, Yalelaan 2, 3584 CM Utrecht, The Netherlands; 3 National Institute for Public Health and the Environment (RIVM), Bilthoven, The Netherlands; 4 Netherlands Institute for Health Services Research (NIVEL), Utrecht, The Netherlands; 5 Faculty of Veterinary Medicine, Utrecht University, Utrecht, The Netherlands

## Abstract

**Background:**

The home address is a common spatial proxy for exposure assessment in epidemiological studies but mobility may introduce exposure misclassification. Mobility can be assessed using self-reports or objectively measured using GPS logging but self-reports may not assess the same information as measured mobility. We aimed to assess mobility patterns of a rural population in the Netherlands using GPS measurements and self-reports and to compare GPS measured to self-reported data, and to evaluate correlates of differences in mobility patterns.

**Method:**

In total 870 participants filled in a questionnaire regarding their transport modes and carried a GPS-logger for 7 consecutive days. Transport modes were assigned to GPS-tracks based on speed patterns. Correlates of measured mobility data were evaluated using multiple linear regression. We calculated walking, biking and motorised transport durations based on GPS and self-reported data and compared outcomes. We used Cohen’s kappa analyses to compare categorised self-reported and GPS measured data for time spent outdoors.

**Results:**

Self-reported time spent walking and biking was strongly overestimated when compared to GPS measurements. Participants estimated their time spent in motorised transport accurately. Several variables were associated with differences in mobility patterns, we found for instance that obese people (BMI > 30 kg/m^2^) spent less time in non-motorised transport (GMR 0.69–0.74) and people with COPD tended to travel longer distances from home in motorised transport (GMR 1.42–1.51).

**Conclusions:**

If time spent walking outdoors and biking is relevant for the exposure to environmental factors, then relying on the home address as a proxy for exposure location may introduce misclassification. In addition, this misclassification is potentially differential, and specific groups of people will show stronger misclassification of exposure than others. Performing GPS measurements and identifying explanatory factors of mobility patterns may assist in regression calibration of self-reports in other studies.

**Electronic supplementary material:**

The online version of this article (doi:10.1186/s12942-017-0103-y) contains supplementary material, which is available to authorized users.

## Background

Environmental epidemiological studies aim at evaluating risks to human health from environmental exposures. Human mobility may affect exposure of persons to different environmental substances, especially if exposure levels display strong spatial, or spatio-temporal variation. Examples of such exposures are ultrafine particles of air pollution [1], electromagnetic fields [2] or livestock-associated exposures, such as zoonotic micro-organisms and endotoxins [3–6]. Personal exposure is often approximated by assigning exposure levels on a single location—usually the home address—to study participants, although this may lead to misclassification of exposure. Exposure misclassification can bias risk estimates, and this bias is often towards the null, in particular when misclassification is non-differential [7–10]. This essentially means that health effects from environmental exposures may remain undetected.

**Fig. 1:**
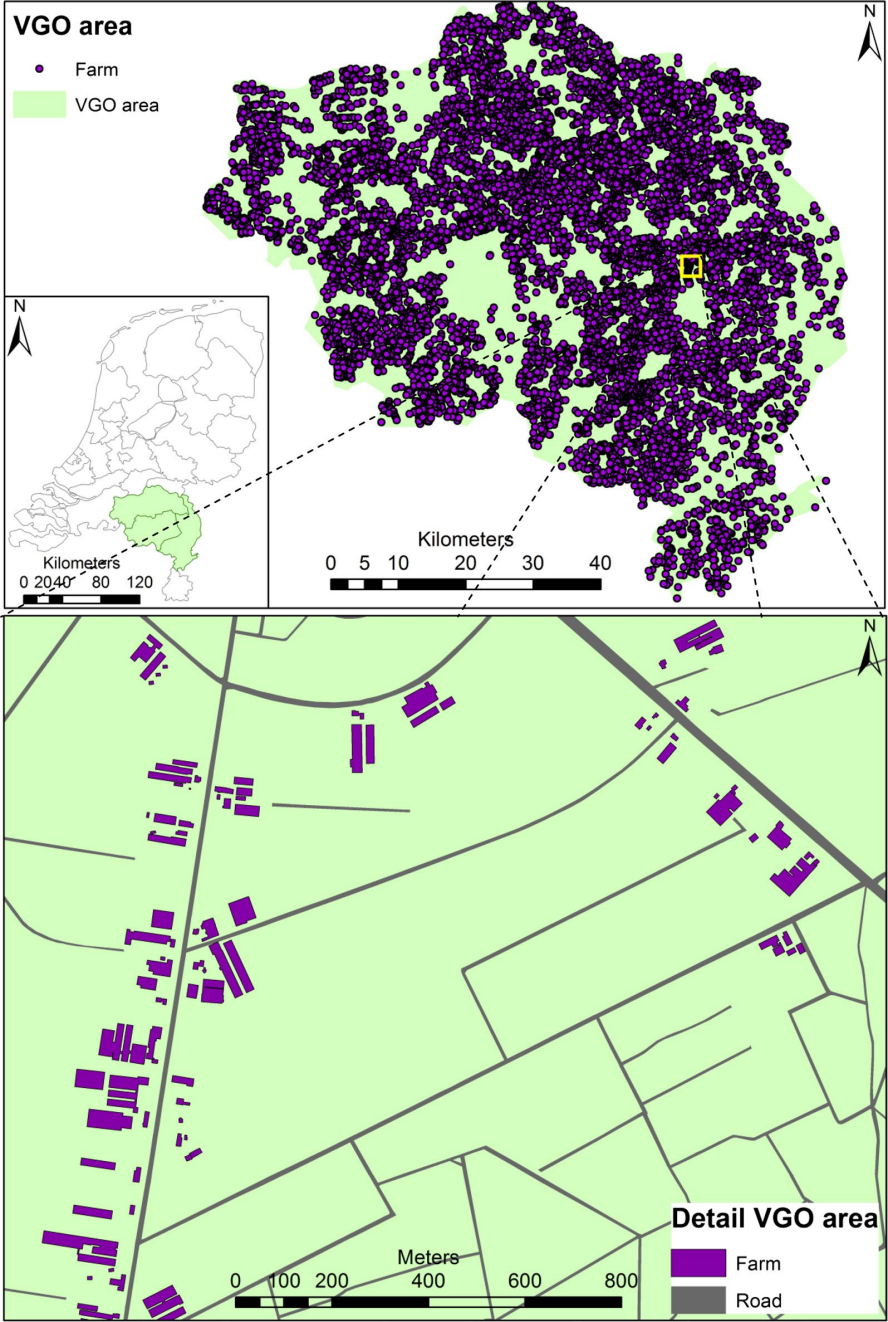
The research area, this map illustrates the rural situation within our research area. Not only are there many farms present in our research area (‘VGO area’ map) these farms are also very close together, with multiple farms per kilometre close to roads <50 m (‘Detail VGO area’ map)

In this study we assessed modes of transport, in particular the duration people spent in motorised or non-motorised transport, and the distance from home for these movements. Mobility patterns can be assessed in multiple ways, using e.g. questionnaire data [11–14] or time activity diaries [14, 15]. Since the 1990s, Global Positioning Systems (GPS) are available that allow for objective measurement of a persons’ movements [16–18]. Measurements with GPS devices and activity diaries are time consuming and thus, questionnaires to assess mobility are often still the method of choice when studying large groups of people. However, self-reports of mobility assessed with questionnaires may be subject to bias and misclassification [11–14], especially if participants answer in a socially desirable way [19, 20]. In addition, the majority of studies addressing mobility are performed among city dwellers [14]. Living in a rural area is likely associated with different mobility patterns [21] and also with different exposures to area-specific emissions, e.g. from livestock farms in the vicinity (Fig. 1). Furthermore, people living in rural areas might spend more time outdoors [21].

In the present study, the main aim was to assess the different modes of transport of a rural population in the Netherlands using GPS measurements. Secondary aims were to explore if we could identify characteristics that explained differences in patterns of transport modes between participants, and to compare self-reported mobility to GPS measured mobility patterns.

## Methods

### Study population

The current study was embedded in the Dutch “Livestock Farming and Neighbouring Residents’ Health Study” (Dutch acronym; VGO). The VGO study focusses on the health of non-farmer residents living in an area with a high density of livestock farms in the Netherlands. In a population-based cohort of 2494 participants (farmers were excluded a priori) [22], a medical examination was conducted by trained fieldworkers (March 2014–February 2015) [23] General Practitioners’ (GPs) Electronic Medical Records (EMRs) were available for 2426 participants (97%) via the Netherlands Institute for Health Services Research (NIVEL, see also http://www.nivel.nl/en), one of the partners in the VGO study. Assessment included a questionnaire (VGO questionnaire) on health, lifestyle factors and the participants’ occupational and residential history. NIVEL provided, when VGO participants gave permission, information regarding asthma, history of heart diseases and beta-blocker usage. VGO cohort members who agreed to be invited for follow-up research were eligible to participate in the GPS study. Medical Ethical approval was obtained for the VGO study from the Medical Ethical Committee of the University Medical Centre Utrecht (protocol number 13/533).

### Study design

From September 2014 to January 2016, eligible subjects were invited to participate in the GPS study. This means that while some participants used GPS loggers in the winter, others used it in the summer. Our dataset therefore pertains to a whole year sample across all seasons. Participants filled in a questionnaire (Q1, see Additional file 1: 11. Questionnaire (Q1)) that inquired about participants’ usual mobility habits regarding different transport modes and time spent outdoors during a regular week. Upon return of Q1, GPS trackers and a second questionnaire (Q2) were sent to participants, including instructions on how to carry the GPS logger for 7 consecutive days. Participants were asked to put the GPS logger next to their keys, in their bag or jacket, so they would not forget it when they left the house. After the GPS-measurement week, Q2 about study adherence and start and end dates of GPS tracker carriage was filled in and GPS loggers were returned to the study centre.

### GPS data

We used TracKing Key Pro GPS loggers (Land Air Sea systems Woodstock IL, USA). These devices enable continuous logging at 1-s intervals. GPS loggers are equipped with a motion sensor, providing data logging only when a participant is moving, thus reducing battery depletion. We set our measurements to 1 s measurement intervals, and the median total logging duration was 187 h (IQR 143–235 h). Data obtained from GPS loggers were date, time, X and Y coordinate and speed (km/h). These GPS loggers were previously tested and showed a high positional accuracy when being outdoors [18].

### Questionnaire data

Q1 included items regarding usual duration of time spent outdoors (hours per day) during the week and weekend, occupational status (being employed/self-employed: yes/no), working from home (yes/no), working days (number), having an outdoor occupation (yes/no), number of outdoor working hours (hours per workday) and outdoor activities during leisure time (walking, biking, sports, spending time close to home, other, in hours per week). Furthermore, transport modes for commuting were asked separately for transport during work hours and during leisure time. Transport modes were stratified by spring/summer, autumn/winter and additionally divided into the sub-categories public transport, car, moped/motorcycle, electric bike, bicycle, on foot and other transport modes. Duration of these transport times was provided in minutes per day for commuting and work-related transport, and in minutes per week for leisure–time transport, participants could report multiple travel modes per trip, therefore alternating mobility patterns should have been captured (Additional file 1: 11. Questionnaire (Q1), an English translation of Q1).

Q2 inquired whether and when participants had left the GPS logger at home during the measuring period and if people had deviated from their normal weekly movement patterns.

Additional participant characteristics and potential explanatory factors for differences in mobility patterns (gender, age, educational level, job status, dog and livestock ownership, hay fever, BMI (measured), smoking status, asthma status, COPD status (self-reporting combined with spirometry data from VGO health survey) and cardiovascular health (recent heart attacks, arrhythmia, ill heart functioning and beta-blocker usage) were obtained from the VGO health assessment and the VGO baseline questionnaire completed at the time of the health assessment (March 2014–February 2015) [22, 23].

### Meteorological data

Meteorological data on precipitation and temperature over the whole measurement period were retrieved from the Royal Netherlands Meteorological Institute. Data from the weather station Eindhoven was used, because this was the most centrally located station of the study area [24]. Percentage of time with rainfall (between 6.00 and 22.00 h) and the average temperature were calculated for the measurement period of each participant.

### Data cleaning

**Fig. 2:**
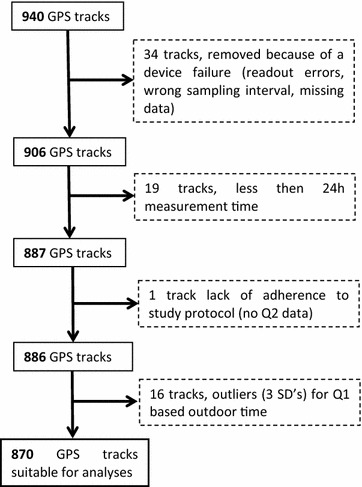
Data cleaning flowchart

**Fig. 3:**
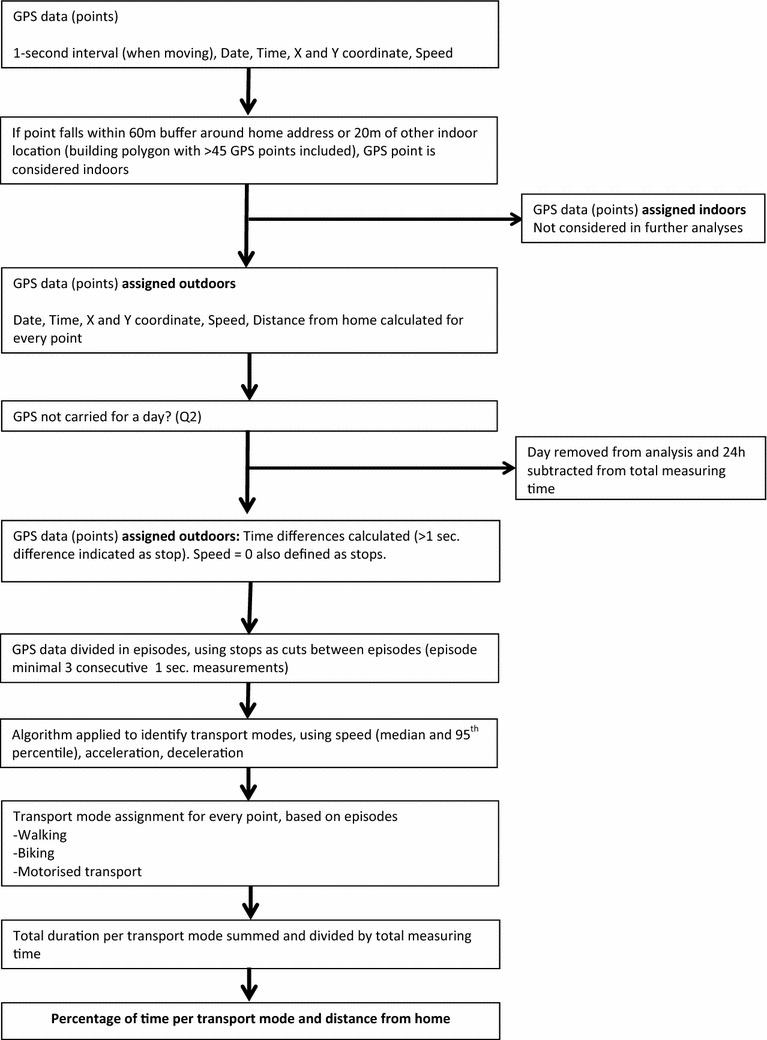
Schematic of GPS processing

We received GPS files from 940 participants. Of these, 34 had to be excluded due to device failure. Two participants did not adhere to the study protocol in that they either did not carry the GPS or did not fill in Q2. In addition, we applied two exclusion criteria: First we excluded persons who had carried the GPS for less than 24 h (N = 19) and second, we excluded persons where the self-reported outdoor time exceeded 3SD of the study population (N = 16). Excluded people reported >64% of their time as being outdoors, which we considered as unrealistic extreme values. One person did not return Q2 and was therefore excluded as well (Fig. 2). In addition, if a participant indicated in Q2 that they had not carried the GPS logger for a specific day, this day was removed from the analyses. More detailed information is provided in Fig. 3. Note that excluded participants did not differ strongly regarding general characteristics (age, sex, education level), compared to participants who remained in the analyses.

### Processing of spatial data

Home addresses (street, postal code, address) were geocoded using Dutch cadastral data (BAG data). A drawback of GPS-tracking is loss of accuracy when a GPS tracker has no clear view of the sky, especially when being indoors [18] resulting in a point cloud (Additional file 1: 1. Example pictures for spatial analysis, Supp. Figure 1). Therefore, point clouds around the home were filtered by excluding all coordinates logged within a 60 m radius around a home location; this distance was based on visual inspection of point clouds around a range of home addresses. Other GPS measurements were classified as indoors when at least 45 points were located within the outline of a building polygon. These polygons were then supplied with a 20 m buffer and all points within this buffer were classified as indoors for further analyses. Again, this cut-off was based on visual inspection: Fewer than 45 indoor points were more likely to appear as linearly-ordered points, indicating smaller spatial inaccuracies when passing a building (Additional file 1: 1. Example pictures for spatial analysis, Supp. Figure 2), while cloud patterns of coordinates were more likely indicating indoor locations, and were often located in public buildings such as sports facilities or supermarkets.

For every point the time differences with the previous point was calculated, if the difference was more than 1 s or speed was 0 km/h, then the point was indicated as a stop. These stops were then used to separate individual mobility episodes. The speed profile of each episode was analysed using a previously developed algorithm that assigns type of transport mode to speed patterns, based on a combination of speed, acceleration and deceleration [25]. Three types of transport modes were assigned to speed profiles: walking, biking or motorised transport. For each transport mode, total duration was assessed and was divided by the total tracking time, resulting in the percentage of time spent per specific transport mode. We analysed our data on a 24 h scale, this means we aimed to evaluate on average 168 h (24 × 7) per participant. Distances from the home address were calculated for each GPS coordinate, by calculating the distance between the GPS coordinate and the border of the 60 m buffer around the home address. Figure 3 shows a schematic of GPS processing.

### Processing of Questionnaire data

In Q1 we asked for mobility per season (spring/summer and autumn/winter), the reported durations for these seasons were linked to the seasons in which participants performed the GPS measurement, the months October–March were considered as autumn/winter and April–September as spring/summer. We expressed data from Q1 pertaining to self-reported transport modes in percentages of time spent per week. Time spent outdoors was calculated by adding the durations for all reported transport modes (commuting, work-related and leisure time) together with time involved in outdoor activities. To compare questionnaire and GPS datasets, time spent outdoors close to home (e.g. gardening, house hold duties, child care, etc.) was subtracted from the total reported time outdoors, as by removing all points within 60 m around a place of residence, we were not able to differentiate erroneous GPS locations from time spent outdoors in close proximity to the home.

### Statistical analysis

Participants were first assigned to an outdoors group based on tertiles of time spent outdoors as provided from their Q1 responses and GPS data [‘little’ (Q1 ≤9.5%, GPS ≤2.4% of time), ‘sometimes’ (Q1 9.5–17.5%, GPS 2.4–4.2% of time) and ‘often’ outdoors (Q1 >17.5%, GPS >4.2% of time)], see Additional file 1: 5. Percentages of time spent outdoors, for distributions of time spent outdoors. They were subsequently assigned to an outdoors group based on identical cut-off values using the tertiles derived from GPS measurements. Cohen’s kappa analyses were then used to compare self-reported data with GPS measured categories of time spent outdoors.

We evaluated six different models with the following dependent variables: percentage of time spent outdoors, percentage of time spent in non-motorised and in motorised transport, mean distance from home while walking, biking and in motorised transport. We chose these outcome variables because they might be interesting for exposure assessment in future studies and differences in exposure due to walking, biking and motorised transport have been analysed extensively before [26].

The following factors were used in the models as independent variables, these were a priori expected to influence time spent outdoors in active transport modes negatively: Chronic Obstructive Pulmonary Disease (COPD) [27], asthma [28], previous heart diseases [29, 30], higher Body Mass Index (BMI) [classified as being overweight (>25–30 kg/m^2^) or obese (>30 kg/m^2^)] [31–33], current smoking [32] and having any symptom in a broad spectrum of health symptoms (Additional file 1: 2. Data used for explanatory variable analysis, Supp. Table 1, and 12. Items from VGO study questionnaire (VGO questionnaire)), attributed to the presence of livestock in the vicinity [34]. In contrast, we expected former and never smokers and people using beta-blockers to be more physically active, the latter on doctors’ advice [35]. We also evaluated whether age (<45, 45–55, 55–65 and >65 years, see Additional file 1: 3. Age distribution of participants in VGO GPS study, Supp. Figure 3, for an age distribution), gender, educational level (low, medium, high) [30], working status (job: yes/no), having an outdoors occupation and the number of workdays per week, were associated with mobility patterns [36]. Furthermore, we expected that people were more frequently outdoors if they reported more time spent outdoors close to home (hours per week) [37], owning a dog (yes/no) [38, 39] or keeping hobby farm animals (yes/no) [37]. The influence of weather conditions, namely average temperature during the measuring period (<5, 5–10, 10–15 (reference group), 15–20, 20–25, >25, all in °C, see Additional file 1: 4. Distribution of avarage temperature during GPS measuring period, Supp. Figure 4, for a temperature distribution) and average rainfall during the measuring period (percentage of time with rainfall between 6.00 and 22.00 h, during measurement) were also evaluated.

Univariate linear regression analyses were performed, followed by multiple linear regression with full models that included all possible explanatory factors for differences in time spent outdoors and distances from home, we used log-transformed data, since data was log normally distributed (data not shown). Supervised stepwise backwards selection (SSBS) models, always including age, gender and educational level, were performed in R. Final SSBS models were selected on the basis of the lowest Akaike’s Information Criterion (AIC). Additional file 1: 6. Supplementary Table 2 (percentage of time) and 7. Supplementary Table 3 (distances from home address) display model outcomes with back transformed coefficients and associated 95% Confidence Intervals (CI), which can be interpreted as Geometric Mean Ratios (GMR) [40]. Finally, we performed sensitivity analyses (Additional file 1: 8. Buffer sizes around the home address, 60 m buffer versus 20 m buffer, Supp Figure 6 and Supp. Table 4) on indoor buffer sizes, using 20 m instead of 60 m buffers around the home address. No substantial differences were observed for measured times spent outdoors (Additional file 1: Supp. Table 4) and therefore, the initial 60 m buffers were retained for all analyses. In Q2 we asked whether people had deviated from their normal weekly movement patterns since this can affect our SSBS model estimates. We ran a sensitivity analyses of our SSBS models by running the models using only participants that indicated to have had a ‘normal week’. Overall we found no material effects on our model estimates (Additional file 1: 9. Supplementary Table 5 and 10. Supplementary Table 6) and therefore preferred to report on our full study population.

Spatial data was processed using ArcGIS ArcMap 10.2 (ESRI, Redlands, CA, USA), statistical analyses were performed using R 3.2.3. (R Foundation, Vienna, Austria).

## Results

From September 2014 to January 2016, 1517 individuals were invited, 1001 (66.0%) agreed to participate in the VGO GPS study and were sent a GPS tracker. A total of 940 GPS tracks contributed to the current analyses, since not all GPS trackers were returned, and 870 tracks remained after data cleaning steps (Fig. 2). The median total GPS measurement duration of all participants was 187 h (IQR 143–235 h), no movement was detected for median 180 h (IQR 136–228 h) and movement was registered for median 6 h (IQR 4–8 h).

Mean age of the participants was 57 years (range 20–72 years), 45% were male and 68% were employed or self-employed. Characteristics of participants are provided in Table 1. Based on GPS data, participants spent a median of 5.5 h/week outdoors: 0.3 h/week walking, 1.1 h/week biking and 3.0 h/week in motorised transport. Median distance from home was 2.0 km for walking (IQR 0.7–7.0), 2.0 km for biking (IQR 0.8–4.4) and 7.4 km for motorised transport (IQR 4.1–14.3) (Table 2).

**Table 1:** General characteristics of study population

Variable	Participants
Total respondents in data analysis (N)	870
Age^b^ [mean, (range)]	57.0 (20.4–72.0)
Sex^b^ [N males, (%)]	391 (44.9)
Education level^b^: low [N (%)]	217 (24.9)
Medium [N (%)]	391 (44.9)
High [N (%)]	262 (30.1)
Job status^a^ [N, working (%)]	592 (68.0)
Number of workdays per week^a^ (mean, range)	2.1 (0-7)
Working from home^a^ [N (% of people with job)]	144 (24.3)
Outdoor occupation^a^ [N (% of people with job)]	70 (11.8)
Outdoor occupation^a^ [Hours per day(mean, range)]	4.6 (1–16)

Data obtained from Q1 (a) and VGO baseline questionnaire (b) [22, 23]

**Table 2:** Data obtained from the GPS track and Q1

Variable	Time in hours/week, distances in km	
	GPS	Questionnaire
Time indoors [Median (IQR)]	162.5 (159.8–164.5)	146.0 (133.9–154.2)
Time outdoors [Median (IQR)]	5.5 (3.5–8.2)	22.0 (13.8–34.1)
Time walking [Median (IQR)]	0.3 (0.1–0.8)	4.0 (2.0–9.0)
Time biking [Median (IQR)]	1.1 (0.3–2.4)	3.0 (1.0–8.0)
Time in motorised transport [Median (IQR)]	3.0 (1.4–5.2)	3.5 (1.8–6.6)
Distances from home while walking [Median (IQR)]	2.0 (0.7–7.0)	
Distances from home while biking [Median (IQR)]	2.0 (0.8–4.1)	
Distances from home motorised transport [Median (IQR)]	7.4 (4.1–14.3)	

Time values are transformed into hours per week, distances are in km from the home address, distance values were only available from the GPS measurements. Time outdoors is a combination of time walking, time biking, time in motorised transport and other time outdoors

**Fig. 4:**
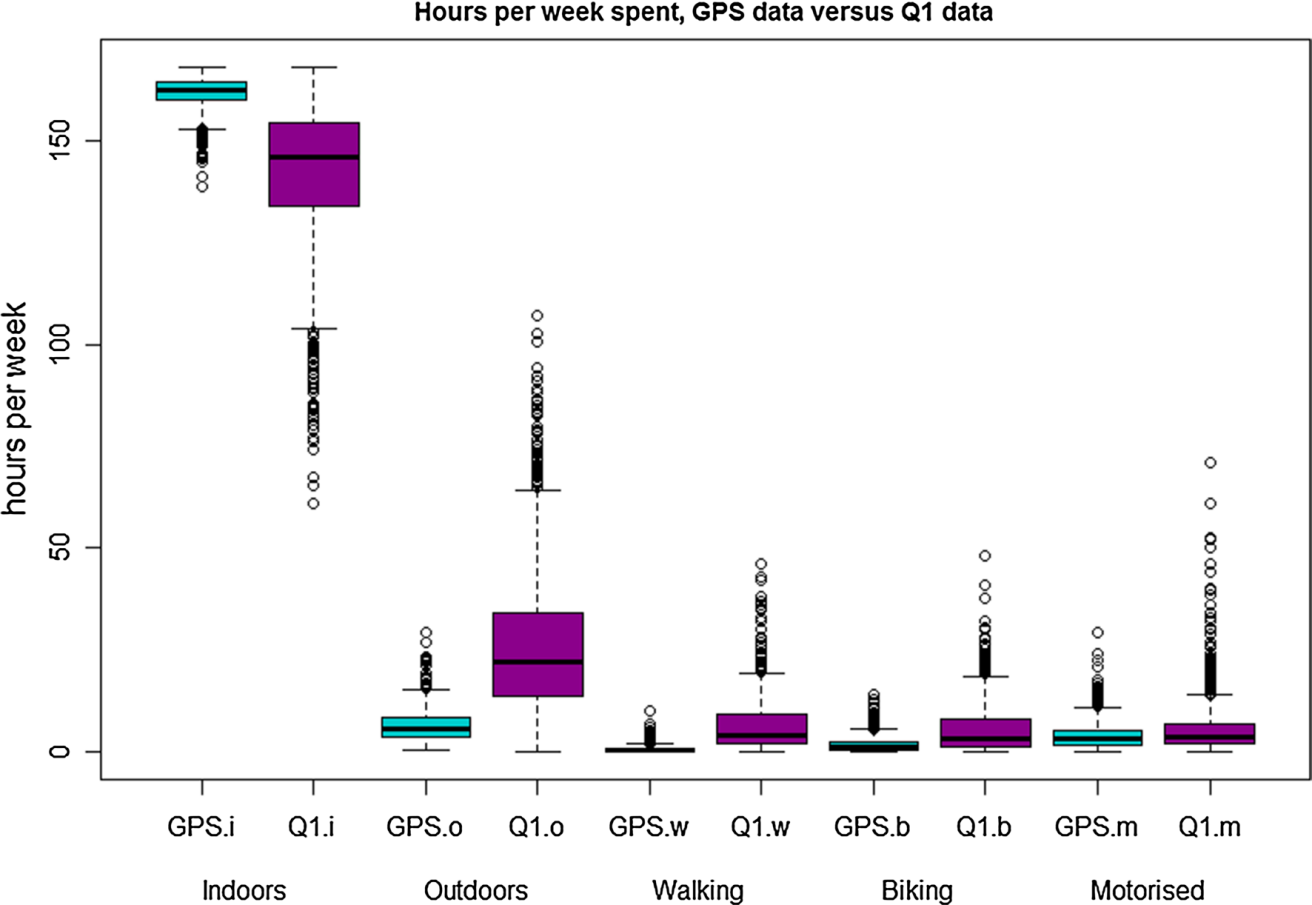
*Boxplots* for hours per week spent: indoors, outdoors, walking, biking and in motorised transport for GPS (*blue*) and Q1 (*purple*) data. Medians and interquartile ranges are provided in Table 2, these *boxplots* illustrate the great differences between GPS measured and self-reported data

The (Q1) reported time spent outside was considerably longer compared to GPS measured time spent outside, indicating substantial overestimation (median 4.0 times longer). Especially walking and biking durations were longer based on self-reported compared to GPS measured durations (median 13.7 and 2.8 times overestimated, respectively), while time spent in motorised transport was similar (median 1.2 times higher), see Table 2 and Fig. 4. The Cohen’s kappa analyses showed a very low agreement between self-reported and measured time spent outdoors (kappa of 0.09 and 0.01, based on tertiles in GPS and Q1 data, and for using the same cut-off values of GPS data to categorise self-reported data, respectively).

Results of our models evaluating individual characteristics on GPS measured mobility patterns are provided in the Additional file 1: Supplementary Tables 2 (percentages of time) and 3 (distances from the home address). Given the discrepancy of self-reports and GPS-measured information, we refrained from evaluating correlates of self-reports.

For the overall percentage of time spent outdoors, cold average temperatures during the measurement period (below 5 °C) was associated with spending less time outdoors (GMR 0.80–0.81), women spent less time outdoors compared to men (GMR 0.85–0.87). People owning a dog spent more time outdoors compared to non-dog-owners (GMR 1.15–1.16).

Compared to study participants with a low educational level, participants with medium or high educational level tended to use motorised over non-motorised transport. We found that obese people (BMI > 30 kg/m^2^) spent less time in non-motorised transport (GMR 0.69–0.74) and people with more workdays spent more time in motorised transport (GMR 1.06–1.12).

Regarding distances from home while walking we observed that higher educated people tended to walk further away from their home (medium educational level GMR 1.31–1.51, high educational level GMR 1.54–1.93), while owning a dog decreased the distance walked from home (GMR 0.51–0.58).

People using beta-blockers walked and biked less far from home than people not using these drugs (walking GMR 0.60–0.71, biking GMR 0.60–0.63). Dog-owners also remained closer to the home while biking, compared with non-dog-owners (GMR 0.73–0.76).

People with COPD and people with more workdays tended to travel longer distances from home in motorised transport (GMR 1.42–1.51 for people with COPD and GMR 1.06–1.09 for each workday). Higher outdoor temperatures (20–25 °C) were associated with shorter distances travelled in motorised transport.

## Discussion

We assessed mobility of a rural population of 870 persons in the Netherlands and found that participants significantly overestimated their time spent outdoors in active transport when self-reported data pertaining to “usual mobility patterns” was compared to GPS measured data. In addition, there was low agreement between self-reported and measured categories of low, medium or high amount of time spent outdoors in active transport (kappa of 0.09). Finally, we identified a range of (participant) characteristics that were associated with differences in mobility patterns of our study population.

### Strengths

Strengths of our study include the large dataset of GPS-measured as well as self-reported mobility patterns. To the best of our knowledge, there are few previous studies with such extensive datasets. Most studies that focus on GPS measurements included fewer than 300 participants [14, 41]. Few larger studies with GPS measurements (Schuessler and Axhausen 2008 N = 4882 and Bohte and Maat 2009 N = 1104) [42, 43], did not evaluate characteristics that explain observed differences in mobility patterns. Our study was embedded in a larger ongoing cohort study, providing additional information for all participants including health data, work and leisure time activities and data about the socio-economic situation of all participants. This extensive dataset enabled us to explore correlates of a range of individual characteristics with mobility patterns of our rural study population.

### Limitations

GPS data has been suggested to add to environmental epidemiological studies, because exposures with a high spatial variability may be more accurately assessed [18]. This is certainly true in the case of GPS logging while in clear view of the sky; in this case, spatial accuracy has been reported to be very high (~2.5 m) [18, 44]. However, when a GPS is used indoors, the spatial accuracy of the measurements is strongly reduced [45]. Therefore, we used buffers around indoor locations to assign these points as being indoors. This procedure thus clearly does not capture all aspects of mobility, but mobility close to home may have gone undetected. Note, however, that applying differently sized home buffers to differentiate indoor from outdoor points did not strongly affect our results. We used GPS measurements as a ‘gold standard’, although GPS measured locations can also have errors. However, we knew from previous work that in general, the accuracy is very high (<10 m) in 85% of the time even when used in an urban area [18]. Since we performed our study in a rural area, with less high-rise buildings, we expected that GPS positional error would not have a significant effect on our findings. Nevertheless, our inability to correctly differentiate measured locations to being either inside or in close proximity to the home likely misclassifies time spent in gardens as indoors. Other researchers have attempted to avoid this spatial accuracy problem by combining GPS measurements with other measurements, such as temperature [46] or a combination of accelerometer, magnetometers and light and temperature sensors [47]. Such a procedure may however increase problems with study adherence if participants have to carry multiple devices, in addition to generating further data analysis complexity.

Another limitation of our study is that we do not have repeated GPS measurements and that participants were only monitored for 1 week. Mobility patterns may change over time, and vary especially with season and weather conditions, as found across our study group. However, we were unable to evaluate whether there are individual differences in the adaptation of mobility patterns to weather or season.

Finally, in our study protocol, we inquired about “usual” daily mobility and not about the actual mobility patterns that participants had followed during our measurement week. We tried to improve match of self-reported and measured data by additionally asking whether participants had deviated from their “usual” weekly mobility patterns in Q2. We found no material differences in the correlates of mobility patterns in a sensitivity analysis of participants who had not deviated from a usual week compared to the full population. Nevertheless, this temporal mismatch may have further contributed to observed variance between self-reports and measured values.

### Comparison self-reported and GPS measured mobility

We observed a striking overestimation in self-reported compared to measured time spent outdoors. Total time spent outdoors might be underestimated since we filtered out GPS locations in a 60 m buffer around the place of residence and 20 m of other indoor locations. In particular time spent walking was significantly overestimated. While overestimation of self-reported time spent walking as such is in line with previous reports, the amount of overestimation is not [14]. Kelly et al. performed a systematic review quantifying differences between self-reported and GPS-measured journey durations. Fourteen publications were included in the meta-analysis and self-reported trip durations were overestimated in all included studies when compared to GPS measurements, overestimations ranged from 9.2 to 75.4% [14]. In our analysis we found an overestimation of 13.7 times for walking, 2.8 times for biking and 1.2 times for motorised transport, which means that only overestimation for motorised transport is in line with what was reported by Kelly et al. [14]. There are three underlying reasons that may be driving this strong observed overestimation for time spent walking. First, in our questionnaire, we inquired about walking durations across different activities, but we did not clearly ask for walking that was performed exclusively outdoors, but asked instead for walking that was done “travelling for work”. This could have resulted in a conceptual mismatch of self-reported and measured data, especially if a considerable part of daily walking is done indoors, e.g. during shopping for work-related purposes or if walking for work indoors (e.g. as a waiter or cleaner) is perceived as “travelling for work”. However, the contribution of walking time of this question to overall walking time had a median below 1%, and only 9.2% of all participants reported any walking for “travelling for work”. Second, the algorithm we used to assign transport modes used the 95th percentile of speed, acceleration and deceleration. This algorithm described in Huss et al. [25] was the best performing algorithm to assign transport modes to GPS data, with a kappa agreement of 0.95 for assigned versus actual mode of transport. The results reported by these authors were based on mobility of 12 participants, but speed patterns used to assign mobility in our dataset might have had a wider variation. However, the speed patterns while walking, biking or in motorised transport are so distinct that we still expect the algorithm to be able to assign transport modes correctly in the majority of the cases. In addition, our algorithm assigned “stops” when the GPS device was not moving, if these stops occurred outdoors, transport modes were not assigned, further contributing to an underestimation of measured outdoor time. We checked the cumulative duration of outdoor stops for each participant, and encountered a maximum of 3 min over the whole study population. Therefore, we do not expect that the use of the algorithm would have introduced the difference in reported and measured mobility patterns. Third, our rural population walked only very little outdoors, across the whole group we measured a median of just 15 min outdoor walking per week. Very short durations, however, are easily misreported and several of our participants also commented that average weekly durations per activity were difficult to estimate. Over-reporting of walking times in our dataset was indeed much less pronounced in persons who walked more (median 4.6 times over-reporting in the highest tertile of walking duration), compared to persons who walked less. Reasons for our rural population to walk so little may be that in general, distances in rural areas tend to be large and many people may thus choose not to walk at all for their mobility needs. Misreporting walking duration may introduce exposure misclassification in studies that attempt to assign outdoor exposures to these durations and/or locations. However, given the very short durations of walking outdoors, the absolute error in exposure assignment may still be limited. Also duration of biking was over-reported by our participants, which highlights that in general, participants overestimate their own amount of active transport outdoors. Motorised transport may be easier to estimate, especially if linked to a fixed schedule in public transport, or if a large part of motorised transport is regular commuting. In studies with a focus on potentially differential concordance/discordance of reported and logged activity locations this disagreement between self-reported and GPS measured spatial data is not present [48, 49]. However, in the current study our focus was on mobility and activity locations were not evaluated as such.

In several previous studies regarding GPS measurements for assessment of physical activity, the authors have not solely relied on GPS measurements, but have combined these with activity diaries or recall interviews [14, 16–18]. Oliver et al. tested the usage of GPS and accelerometry tools to assess transport-related physical activity (i.e. walking, biking); the comparative standard in this study were questionnaire travel logs. They included 37 participants into their study and concluded that GPS and accelerometry were good tools to assess walking and biking activity, although performance of the questionnaire data was not assessed [19]. Sallis et al. compared interviewer-administered and self-reported questionnaires, heart-rate monitors, and accelerometers for activity patterns of fifth graders. Both questionnaire approaches correlated quite well (Pearson’s r = 0.76) but correlation between questionnaires and objective measurements (heart-rate monitor and accelerometer) was lower (r = 0.50 and r = 0.30, respectively) [50]. These effects can partially be explained with a tendency to answer in a socially desirable way, resulting in over-reporting of activity durations, as shown by Adams et al. [20]. This means that regression calibration using measurements (GPS or mobile phone data) performed in a subsample of study participants may represent a way to calibrate self-reports [51], although this approach has not been validated in different populations.

### Explanatory variables analyses

To the best of our knowledge we are the first to identify several correlates of mobility patterns, which may be especially relevant when assessing exposure to agents with a high spatial variability. For example, certain emissions from livestock farms are only detectable at a short distance: detectable levels of viable organisms have been found between 150 and 160 m from pig stables [4, 52] and at 330 m from poultry stables [3]. Even higher spatial variability can be observed for other environmental exposures, such as particulate matter [53] or electromagnetic fields [2]. This means that if mobility is relevant for personal exposure levels, using a general approach such as assigning exposure to the home address, will misclassify specific groups of people more than others. The identified individual explanatory factors for differences in mobility patterns may thus further assist in regression calibration efforts for other studies, or in the interpretation of previous studies that did not take such explanatory factors into account.

## Future perspectives

Until very recently, due to financial, logistic and data management limitations, GPS measurements were only used in a limited way for data collection in mobility assessment. When GPS measurements were collected, this was generally done in small samples of people. Self-reporting with all its disadvantages including recall bias [11–14] was the default method to collect movement data on large cohorts of people [14]. With the increasing capabilities of smartphones [1, 54–57], new opportunities exist to gather objectively measured data regarding spatial positions of people. Dewulf et al. illustrated this by combining location data from mobile phone network providers with air pollution data from a monitoring network in Belgium [1]. Using smartphones for location assessment in studies may thus help in reducing the amount of measurement devices a participant has to carry around. It may further assist in upscaling objective measurements to large cohort study collectives. Epidemiological studies relying on self-reports of usual mobility patterns should be aware of possible over-reporting of active transport patterns. Ways to mitigate this include improving temporal matching by using detailed activity diaries instead of asking for “usual” mobility, or possibly to improve reporting by regression calibration methods [58, 59].

## Conclusions

We evaluated mobility of a rural population and found that participants significantly overestimated their time spent outdoors in active transport when self-reported data was compared to GPS measured data. We identified several correlates of mobility patterns, which may be especially relevant when assessing exposure to agents with a high spatial variability. If active transport outdoors is relevant for personal exposure levels, then using a general approach such as assigning exposure to the home address will introduce exposure misclassification that will be stronger in some groups of people than in others. Regression calibration using measurements or these identified explanatory variables may represent a way to calibrate self-reports in future studies.

## Additional file

**Additional file 1.** Supplementary data.

## References

[1] Dewulf B, Neutens T, Lefebvre W, Seynaeve G, Vanpoucke C, Beckx C (2016). Dynamic assessment of exposure to air pollution using mobile phone data. Int J Health Geogr [Internet].

[2] Beekhuizen J, Vermeulen R, Kromhout H, Bürgi A, Huss A (2013). Science of the total environment geospatial modelling of electromagnetic fields from mobile phone base stations. Sci Total Environ [Internet].

[3] Schulz J, Formosa L, Seedorf J, Hartung J (2011). Measurement of culturable airborne staphylococci downwind from a naturally ventilated broiler house. Aerobiologia (Bologna).

[4] Schulz J, Friese A, Klees S, Tenhagen BA, Fetsch A, Rösler U (2012). Longitudinal study of the contamination of air and of soil surfaces in the vicinity of pig barns by livestock-associated methicillin-resistant *Staphylococcus aureus*. Appl Environ Microbiol.

[5] Schulze A, Römmelt H, Ehrenstein V, van Strien R, Praml G, Küchenhoff H (2011). Effects on pulmonary health of neighboring residents of concentrated animal feeding operations: exposure assessed using optimized estimation technique. Arch Environ Occup Health.

[6] Klous G, Huss A, Heederik DJJ, Coutinho RA (2016). Human–livestock contacts and their relationship to transmission of zoonotic pathogens, a systematic review of literature. One Health [Internet]..

[7] Kersh GJ, Fitzpatrick KA, Self JS, Priestley RA, Kelly AJ, Ryan Lash R (2013). Presence and persistence of *Coxiella burnetii* in the environments of goat farms associated with a Q fever outbreak. Appl Environ Microbiol.

[8] Perchoux C, Chaix B, Cummins S, Kestens Y (2013). Conceptualization and measurement of environmental exposure in epidemiology: accounting for activity space related to daily mobility. Heal Place [Internet].

[9] Chaix B, Méline J, Duncan S, Merrien C, Karusisi N, Perchoux C (2013). GPS tracking in neighborhood and health studies: A step forward for environmental exposure assessment, a step backward for causal inference?. Heal Place.

[10] Rytkönen MJ (2004). Not all maps are equal: GIS and spatial analysis in epidemiology. Int J Circumpolar Health.

[11] Stalvey BT, Owsley C, Sloane ME, Ball K (1999). The life space questionnaire: a measure of the extent of mobility of older adults. J Appl Gerontol.

[12] O’Brien M, Jones D, Sloan D, Rustin, M. From the SAGE Social Science Collections. All Rights Reserved. 2016.

[13] Meurs H, Haaijer R (2001). Spatial structure and mobility. Transp Res Part D Transp Environ.

[14] Kelly P, Krenn P, Titze S, Stopher P, Foster C (2013). Quantifying the difference between self-reported and global positioning systems-measured journey durations: a systematic review. Transp Rev A Transnatl Transdiscipl J [Internet].

[15] Deffner V, Kuchenhoff H, Maier V, Pitz M, Cyrys J, Breitner S (2016). Personal exposure to ultrafine particles: Two-level statistical modeling of background exposure and time-activity patterns during three seasons. J Expo Sci Environ Epidemiol [Internet].

[16] Elgethun K, Fenske RA, Yost MG, Palcisko GJ (2003). Time-location analysis for exposure assessment studies of children using a novel global positioning system instrument. Environ Health Perspect.

[17] Dias D, Tchepel O (2014). Modelling of human exposure to air pollution in the urban environment: a GPS-based approach. Environ Sci Pollut Res.

[18] Beekhuizen J, Kromhout H, Huss A, Vermeulen R (2013). Performance of GPS-devices for environmental exposure assessment. J Expo Sci Environ Epidemiol [Internet].

[19] Oliver M, Badland H, Mavoa S, Duncan MJ, Duncan S (2010). Combining GPS, GIS, and accelerometry: methodological issues in the assessment of location and intensity of travel behaviors. J Phys Act Health.

[20] Adams SA, Matthews CE, Ebbeling CB, Moore CG, Joan E, Fulton J (2005). The effect of social desirability and social approval on self-reports of physical activity. Am J Epidemiol.

[21] Matz CJ, Stieb DM, Brion O (2015). Urban-rural differences in daily time-activity patterns, occupational activity and housing characteristics. Environ Health [Internet].

[22] Borlée F, Yzermans CJ, Van Dijk CE, Heederik D, Smit LAM (2015). Increased respiratory symptoms in COPD patients living in the vicinity of livestock farms. Eur Respir J [Internet].

[23] Borlée F, Yzermans CJ, Krop E, Aalders B, Rooijackers J, Zock JP, van Dijk CE, Maassen K, Schellevis F, Heederik D, Smit LAM (2017). Spirometry, questionnaire and Electronic Medical Record based COPD in a population survey: comparing prevalence, level of agreement and associations with potential risk factors. PLoS ONE.

[24] KNMI website [Internet]. https://www.knmi.nl/Nederland-nu/klimatologie/uurgegevens. Accessed 4 April 2016.

[25] Huss A, Beekhuizen J, Kromhout H, Vermeulen R (2014). Using GPS-derived speed patterns for recognition of transport modes in adults. Int J Health Geogr [Internet]..

[26] De Hartog JJ, Boogaard H, Nijland H, Hoek G (2010). Do the health benefits of cycling outweigh the risks?. Environ Health Perspect.

[27] Pitta F, Troosters T, Spruit MA, Probst VS, Decramer M, Gosselink R (2005). Characteristics of physical activities in daily life in chronic obstructive pulmonary disease. Am J Respir Crit Care Med.

[28] Williams B, Powell A, Hoskins G, Neville R (2008). Exploring and explaining low participation in physical activity among children and young people with asthma: a review. BMC Fam Pract [Internet].

[29] Giallauria F, Cirillo P, D’agostino M, Petrillo G, Vitelli A, Pacileo M (2011). Effects of exercise training on high-mobility group box-1 levels after acute myocardial infarction. J Card Fail [Internet].

[30] Allman RM, Baker PS, Maisiak RM, Sims RV, Roseman JM (2004). Racial similarities and differences in predictors of mobility change over eighteen months. J Gen Intern Med.

[31] Kyttä AM, Broberg AK, Kahila MH (2012). Urban environment and children’s active lifestyle: SoftGIS revealing children’s behavioral patterns and meaningful places. Am J Health Promot.

[32] Stuck AE, Walthert JM, Nikolaus T, Büla CJ, Hohmann C, Beck JC (1999). Risk factors for functional status decline in community-living elderly people: a systematic literature review. Soc Sci Med.

[33] BMI [Internet]. http://www.nhlbi.nih.gov/health/educational/lose_wt/BMI/bmi-m.htm.

[34] Neuberg SL, Kenrick DT, Schaller M (2011). Human threat management systems: self-protection and disease avoidance. Neurosci Biobehav Rev [Internet]..

[35] Graham I, Atar D, Borch-Johnsen K, Boysen G, Burell G, Cifkova R (2007). European guidelines on cardiovascular disease prevention in clinical practice: executive summary: fourth joint task force of the European Society of Cardiology and other societies on cardiovascular disease prevention in clinical practice (Constituted by representatives of nine societies and by invited experts). Eur Heart J.

[36] Stopher P, Zhang Y. Is travel behaviour repetitive from day to day? [Internet]. http://atrf.info/papers/2010/2010_Stopher_Zhang_B.pdf, 33rd Australasian Transport Research Forum (ATRF) 2010, Canberra, ACT, Australia.

[37] Bellows AC, Brown K, Smit J (2003). Health benefits of urban agriculture.

[38] Brown JD, Stallknecht DE, Valeika S, Swayne DE (2007). Susceptibility of wood ducks to H5N1 highly pathogenic avian influenza virus. J Wildl Dis.

[39] Cutt H, Giles-Corti B, Knuiman M, Burke V (2007). Dog ownership, health and physical activity: a critical review of the literature. Health Place.

[40] Kytariolos J, Karalis V, Macheras P, Symillides M (2006). Novel scaled bioequivalence limits with leveling-off properties. Pharm Res.

[41] Chaix B, Kestens Y, Duncan DT, Brondeel R, Méline J, El Aarbaoui T, et al. A GPS-based methodology to analyze environment-health associations at the trip level: case-crossover analyses of built environments and walking. Am J Epidemiol [Internet]. 2016; kww071. http://www.ncbi.nlm.nih.gov/pubmed/27659779. 10.1093/aje/kww071 27659779

[42] Schuessler N, Axhausen KW (2009). Processing raw data from global positioning systems without additional information. Transp Res Rec J Transp Res Board.

[43] Bohte W, Maat K (2009). Deriving and validating trip purposes and travel modes for multi-day GPS-based travel surveys: a large-scale application in the Netherlands. Transp Res Part C Emerg Technol [Internet]..

[44] Krenn PJ, Titze S, Oja P, Jones A, Ogilvie D (2011). Use of global positioning systems to study physical activity and the environment a systematic review. Am J Prev Med.

[45] Kerr J, Duncan S, Schipperjin J (2011). Using global positioning systems in health research a practical approach to data collection and processing. Am J Prev Med.

[46] Nethery E, Mallach G, Rainham D, Goldberg MSMS, Wheeler AJAJ (2014). Using Global Positioning Systems (GPS) and temperature data to generate time-activity classifications for estimating personal exposure in air monitoring studies: an automated method. Environ Health.

[47] Matthews CE, Moore SC, George SM, Sampson J, Bowles HR (2012). Improving self-reports of active and sedentary behaviors in large epidemiologic studies. Exerc Sport Sci Rev..

[48] Shareck M, Kestens Y, Gauvin L (2013). Examining the spatial congruence between data obtained with novel activity location questionnaire, continuos GPS tracking, and prompted recall surveys. Int J Health Geogr [Internet].

[49] Paz-Soldan VA, Reiner RC, Morrison AC, Stoddard ST (2014). Strenghts and weaknesses of Global Positioning System (GPS) data-loggers and semi-structured interviews for capturing fine-scale human mobility: findings from Iquitos, Peru. PLoS Negl Trop Dis.

[50] Sallis JF, Strikmiller PK, Harsha DW, Feldman HA, Ehlinger S, Stone EJ, Williston J, Woods S (1996). Validation of interviewer- and self-administered physical activity checklists for fifth grade students. Med Sci Sports Exerc..

[51] Matthews CE, Moore C, George SM, Sampson J, Bowles HR. NIH Public Access. 2013;40(3):118–26. 10.1097/JES.0b013e31825b34a0 PMC3388604 22653275

[52] Thorne PS. Industrial livestock production facilities: airborne emissions. Encycl Environ Heal [Internet]. 2011;218–26. http://www.sciencedirect.com/science/article/pii/B9780444522726004232.

[53] Li J, Jin M, Xu Z. Spatiotemporal variability of remotely sensed PM 2. 5 Concentrations in China from 1998 to 2014 Based on a Bayesian Hierarchy Model. 2014. 10.3390/ijerph13080772 PMC4997458 27490557

[54] Gonzalez MC, Hidalgo CA, Barabasi A-L (2008). Understanding individual human mobility patterns. Nature [Internet].

[55] Ahas R, Silm S, Järv O, Saluveer E, Tiru M (2010). Using mobile positioning data to model locations meaningful to users of mobile phones. J Urban Technol.

[56] Glasgow ML, Rudra CB, Yoo E-H, Demirbas M, Merriman J, Nayak P (2014). Using smartphones to collect time–activity data for long-term personal-level air pollution exposure assessment. J Expo Sci Environ Epidemiol [Internet]..

[57] Palmer JRB, Espenshade TJ (2013). New approaches to human mobility: using mobile phones for demographic Research. Demography.

[58] Lim S, Wyker B, Bartley K, Eisenhower D (2015). Measurement error of self-reported physical activity in New York City: assessement and correction. Am J Epidemiol.

[59] Saint-Maurice PF, Welk GJ, Beyler NK, Bartee RT, Heelan KA (2014). Calibration of self-report tools for physical activity research: the Physical Activity Questionnaire (PAQ). BMC Public Health.

