# Supplementary material for: Mobility assessment of a rural population in the Netherlands using GPS measurements
Source: Int J Health Geogr. 2017 Aug 9;16:30. doi: 10.1186/s12942-017-0103-y (PMC5551017; doi:10.1186/s12942-017-0103-y)
Supplement: Supplementary file 1 — Additional file 1. Supplementary data. [file 12942_2017_103_MOESM1_ESM.doc]

**Additional file 1: Supplementary data**

***1.*** *Example pictures for the spatial analyses*

60m home buffer (Supp. figure 1) and other indoor points (Supp. figure 2).


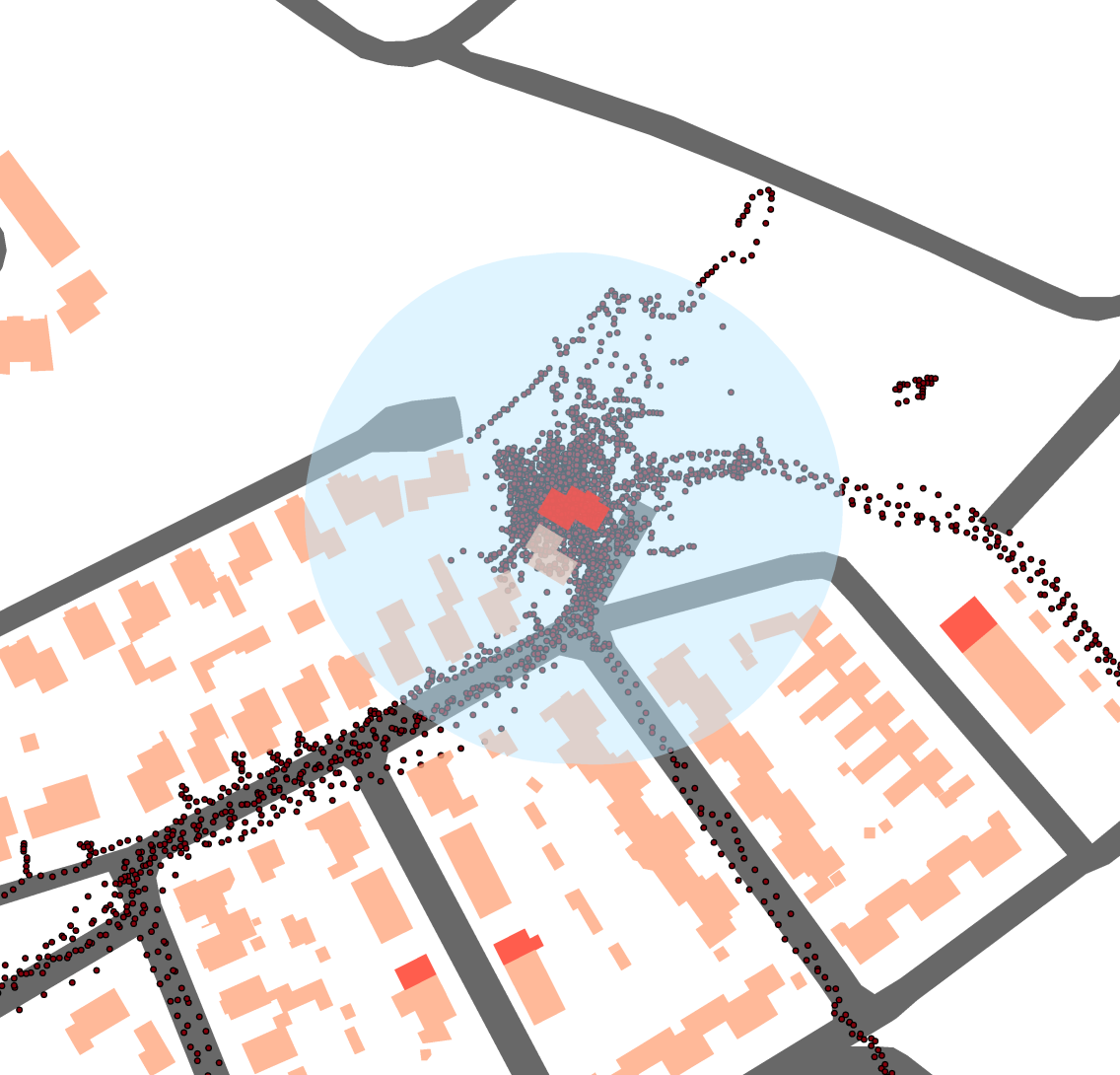


**Supp. Figure 1.** A typical GPS point cloud around a home address (red polygon), this was resolved by using a 60m buffer around the home address (light blue), all GPS points within this buffer were indicated as being ‘indoors’, all points outside this buffer and additional indoor buffers, were indicated as ‘outdoors’ and used in the analyses.


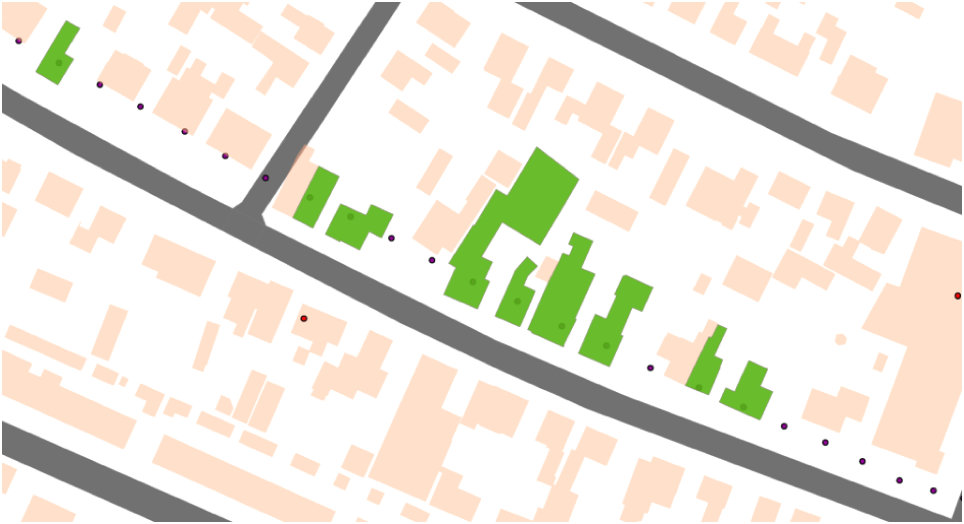
**Supp. Figure 2.** Measurement error in a GPS track (within the red line), based on the shape of the GPS track, this person was driving in a car on the major road (grey), due to the GPS measuring error some of the GPS points fell within building polygons (**green**, for those with a point included, **pink** for building polygons without a GPS point inside). GPS points, outside the home buffer, were only assigned as ‘indoors’ if more than 45 points were located within a building polygon. If this was the case a 20m buffer was used around the specified building to assign those points as ‘indoors’ using a similar approach as with the home buffer (see Supp. Figure 1).

***2.*** *Data used for explanatory variable analyses*

| **Explanatory variable** | **Prevalence (N (%))** | **Data used** |
| --- | --- | --- |
| COPD, from VGO questionnaire | 78 (9%) | Self-reported: ‘Have you ever been told by a doctor that you had chronic obstructive pulmonary disease or emphysema?’  Based on spirometry:   - Post-BD measurement of FEV1/FVC below the lower limits of normal (LLN was calculated with GLI-reference values based on age, gender and height)   AND/OR  - Post-BD measurement of FEV1/FVC <0.70 (GOLD). LLN was calculated with GLI-reference values based on age, gender and height (add. 1, add. 2) |
| Asthma, from VGO questionnaire | 46 (5%) | Self-reported: “did you ever have asthma, and was this confirmed by a doctor?” |
| Heart diseases, from VGO questionnaire | 27 (3%) | Self-reported: ”Are you treated for heart arrhythmia by a cardiologist?” “have you experienced a heart attack in the recent 3 months?”  “do you have a poorly functioning heart?”  grouped as ‘any self-reported heart problems’ |
| People perceiving health complaints from livestock farms, from VGO questionnaire | 67 (8%) | Self-reported: “do you think the health complaints you selected, are possibly linked to livestock farms in your home vicinity?” |
| Outdoors occupation, from Q1 | 70 (8%) | Self-reported, people agreed on the following: “most work-activities are outdoors, and work takes place outdoors for several hours per day” |

**Supp. Table 1.** Data used for specific explanatory variables.


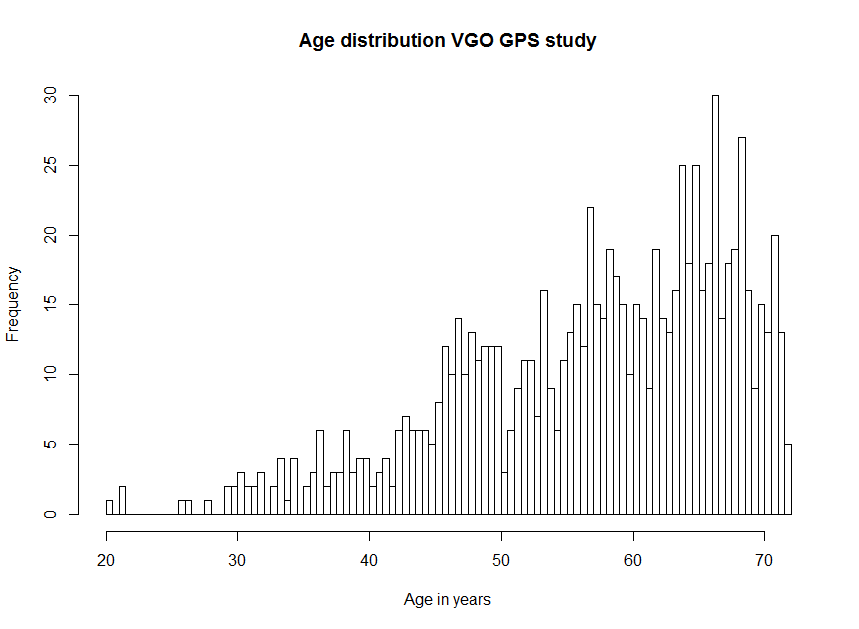
***3.*** *Age distribution of participants in VGO GPS study*

**Supp. Figure 3.** Distribution of age of participants in the VGO GPS study, based on this distribution four age categories were assigned (<45yrs, 45-55yrs, 55-65yrs, >65yrs), these categories were used in the explanatory variables analyses.


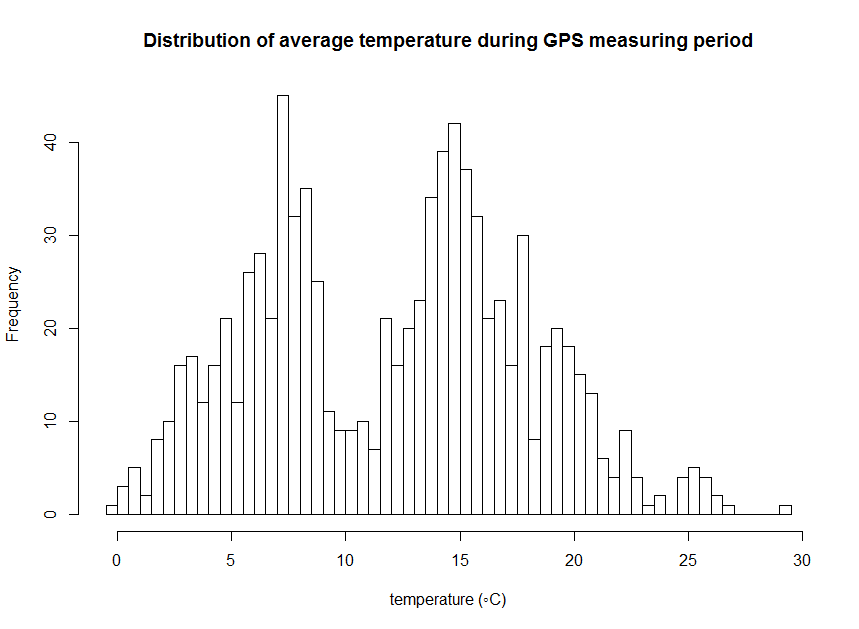
***4.*** *Distribution of avarage temperature during GPS measuring period*

**Supp. Figure 4.** Distribution of average temperature during the GPS measurement. The following categories were assigned (<5°C, 5-10°C, 10-15°C, 15-20°C, 20-25°C, >25°C) the category 10-15°C was chosen as reference category, because this category included both the median (12.9°C) and mean (12.1°C) temperature.


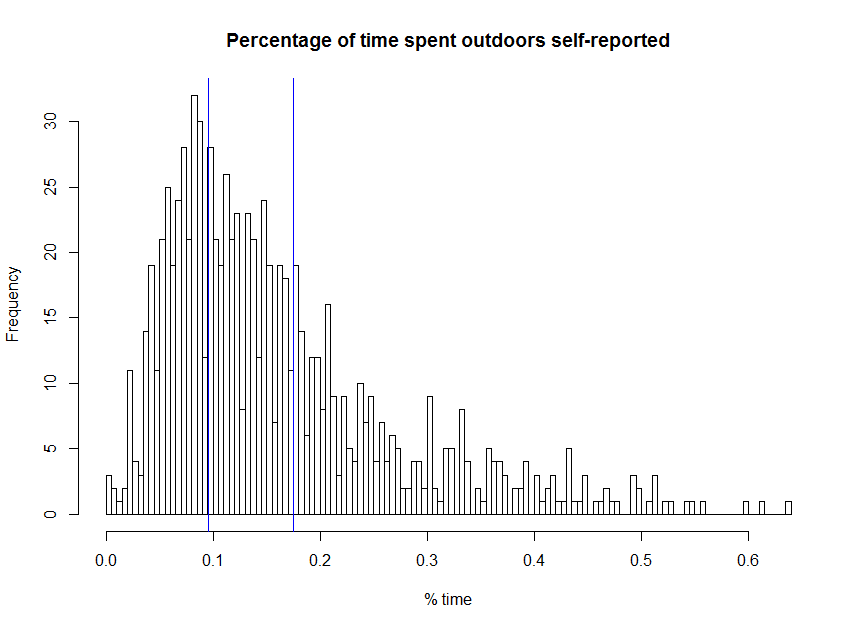

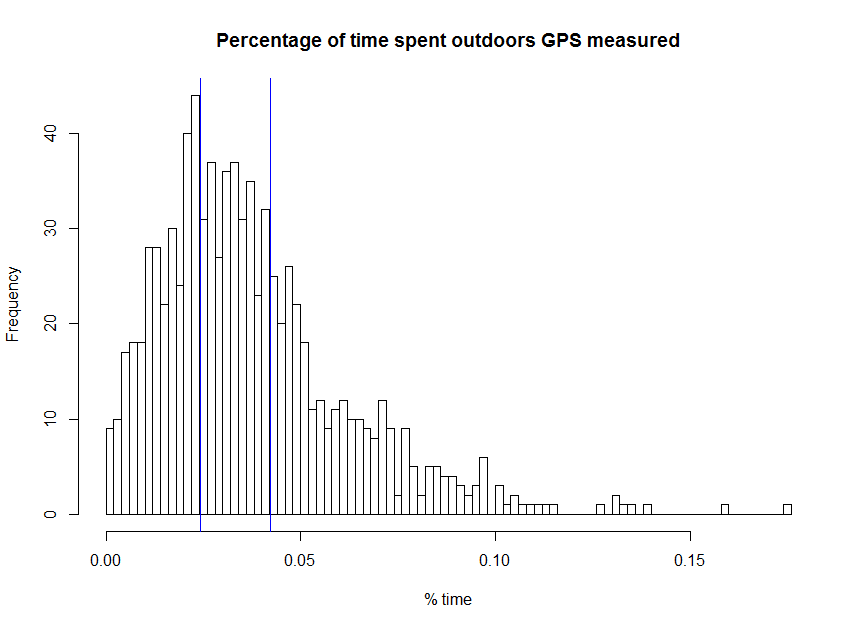


A

B

***5.*** *Percentages of time spent outdoors A. self-reported, B. GPS measured, with cut-offs used in kappa analysis*

**Supp.Figure 5 A and B.** Distributions of percentages of time spent outside, measured with Q1 (A) and GPS (B). The tertiles of the distributions (in blue Q1: 0.095 and 0.175, GPS: 0.024 and 0.042) of these figures provided the cut-off values for the assignment of the outdoors groups used in the Cohen’s kappa analyses.

| Supp. Table 2 Overview model variables  (percentages of time) | | | **Univariate models** | |  | **Full models**  6. Supplementary Table 2 (percentages of time) | |  | **SSBS models** | |
| --- | --- | --- | --- | --- | --- | --- | --- | --- | --- | --- |
| Outcome | Variable | Category | **GMR (95% CI)** | **P.value** |  | **GMR (95% CI)** | **P.value** |  | **GMR (95% CI)** | **P.value** |
| Percentage of time spent outside | Age | 45-55y | 1.12 (0.94-1.34) | 0.20 |  | 1.11 (0.93-1.34) | 0.25 |  | 1.12 (0.94-1.34) | 0.21 |
|  |  | 55-65y | 1.04 (0.88-1.22) | 0.67 |  | 1.04 (0.87-1.26) | 0.65 |  | 1.07 (0.89-1.28) | 0.46 |
|  |  | >65y | 0.96 (0.80-1.14) | 0.61 |  | 0.99 (0.80-1.23) | 0.92 |  | 1.02 (0.83-1.26) | 0.84 |
|  | Gender | Female | 0.85 (0.77-0.94) | <0.01 |  | 0.87 (0.77-0.97) | 0.01 |  | 0.86 (0.77-0.95) | <0.01 |
|  | BMI | Overweight  (25-30 kg/m2) | 1.04 (0.92-1.17) | 0.52 |  | 1.02 (0.91-1.15) | 0.71 |  |  |  |
|  |  | Obese  (>30 kg/m2) | 0.92 (0.79-1.06) | 0.25 |  | 0.90 (0.77-1.05) | 0.19 |  |  |  |
|  | Smoker | Former | 0.96 (0.86-1.07) | 0.48 |  | 0.99 (0.88-1.11) | 0.86 |  |  |  |
|  |  | Current | 0.90 (0.74-1.10) | 0.30 |  | 0.92 (0.76-1.13) | 0.44 |  |  |  |
|  | Education level | Medium | 1.08 (0.95-1.23) | 0.23 |  | 1.07 (0.93-1.23) | 0.33 |  | 1.07 (0.94-1.22) | 0.31 |
|  |  | High | 1.16 (1.01-1.34) | 0.03 |  | 1.15 (0.98-1.33) | 0.08 |  | 1.14 (0.98-1.31) | 0.09 |
|  | Duration of rainfall | (% time over measuring period) | 0.70 (0.30-1.63) | 0.41 |  | 0.72 (0.28-1.83) | 0.49 |  |  |  |
|  | Temperature (average over measuring period) | <5°C | 0.80 (0.67-0.95) | 0.01 |  | 0.81 (0.68-0.97) | 0.02 |  | 0.80 (0.67-0.95) | 0.01 |
|  |  | 5-10°C | 1.01 (0.88-1.17) | 0.85 |  | 1.01 (0.87-1.18) | 0.85 |  | 1.00 (0.86-1.15) | 0.95 |
|  |  | 15-20°C | 0.99 (0.86-1.15) | 0.95 |  | 0.97 (0.84-1.13) | 0.72 |  | 0.98 (0.84-1.13) | 0.74 |
|  |  | 20-25°C | 0.95 (0.76-1.19) | 0.66 |  | 0.92 (0.73-1.16) | 0.48 |  | 0.94 (0.75-1.17) | 0.56 |
|  |  | >25°C | 1.34 (0.86-2.11) | 0.20 |  | 1.35 (0.85-2.13) | 0.20 |  | 1.40 (0.89-2.19) | 0.14 |
|  | Job status | (employed) | 1.06 (0.95-1.18) | 0.31 |  | 0.91 (0.78-1.06) | 0.21 |  |  |  |
|  | Workdays | (days per week) | 1.03 (1.01-1.06) | 0.01 |  | 1.03 (0.99-1.07) | 0.12 |  | 1.02 (0.99-1.05) | 0.17 |
|  | Outdoors occupation | (yes) | 1.19 (0.98-1.44) | 0.07 |  | 1.12 (0.91-1.37) | 0.30 |  |  |  |
|  | COPD | (yes) | 1.00 (0.84-1.20) | 0.97 |  | 1.00 (0.82-1.20) | 0.97 |  |  |  |
|  | Asthma | (doctor diagnosed) | 1.00 (0.99-1.00) | 0.20 |  | 1.00 (0.99-1.00) | 0.17 |  | 1.00 (0.99-1.00) | 0.13 |
|  | Hayfever | (self-reported) | 1.03 (0.92-1.16) | 0.57 |  | 1.05 (0.93-1.18) | 0.47 |  |  |  |
|  | Betablocker usage | (yes) | 0.97 (0.82-1.16) | 0.74 |  | 1.03 (0.85-1.24) | 0.77 |  |  |  |
|  | History of heart diseases | (yes) | 1.23 (0.91-1.66) | 0.17 |  | 1.30 (0.94-1.78) | 0.11 |  | 1.28 (0.94-1.73) | 0.11 |
|  | Person thinks health complaints are due to nearby livestock | (yes) | 1.00 (1.00-1.00) | 0.41 |  | 1.00 (1.00-1.00) | 0.73 |  |  |  |
|  | Time spent outdoors close to home | (hours per week) | 1.00 (0.99-1.01) | 0.94 |  | 1.00 (0.99-1.01) | 0.73 |  |  |  |
|  | Animal ownership | Dog | 1.15 (1.02-1.30) | 0.02 |  | 1.16 (1.02-1.32) | 0.02 |  | 1.15 (1.02-1.31) | 0.03 |
|  |  | Livestock | 1.00 (1.00-1.01) | 0.62 |  | 1.00 (1.00-1.01) | 0.24 |  | 1.00 (1.00-1.01) | 0.15 |
|  | | | | | | | | | | |
| Percentage of time spent in non-motorised  transport | Age | 45-55y | 1.33 (0.99-1.81) | 0.06 |  | 1.25 (0.92-1.70) | 0.15 |  | 1.25 (0.92-1.70) | 0.15 |
|  |  | 55-65y | 1.73 (1.31-2.29) | <0.01 |  | 1.47 (1.08-2.01) | 0.02 |  | 1.43 (1.06-1.95) | 0.02 |
|  |  | >65y | 1.94 (1.45-2.61) | <0.01 |  | 1.43 (0.99-2.06) | 0.06 |  | 1.38 (0.97-1.97) | 0.07 |
|  | Gender | Female | 0.97 (0.81-1.15) | 0.70 |  | 0.98 (0.81-1.19) | 0.85 |  | 0.99 (0.82-1.19) | 0.90 |
|  | BMI | Overweight  (25-30 kg/m2) | 0.99 (0.80-1.21) | 0.89 |  | 0.95 (0.77-1.16) | 0.61 |  | 0.96 (0.78-1.17) | 0.66 |
|  |  | Obese  (>30 kg/m2) | 0.74 (0.57-0.95) | 0.02 |  | 0.70 (0.54-0.91) | 0.01 |  | 0.69 (0.54-0.90) | 0.01 |
|  | Smoker | Former | 1.09 (0.90-1.31) | 0.37 |  | 0.96 (0.79-1.16) | 0.65 |  | 0.93 (0.77-1.13) | 0.49 |
|  |  | Current | 0.66 (0.47-0.93) | 0.02 |  | 0.66 (0.47-0.93) | 0.02 |  | 0.64 (0.46-0.89) | 0.01 |
|  | Education level | Medium | 0.91 (0.73-1.13) | 0.38 |  | 0.96 (0.76-1.20) | 0.70 |  | 0.95 (0.76-1.20) | 0.68 |
|  |  | High | 0.82 (0.65-1.05) | 0.11 |  | 0.91 (0.70-1.18) | 0.47 |  | 0.90 (0.70-1.16) | 0.42 |
|  | Duration of rainfall | (% time over measuring period) | 0.26 (0.06-1.10) | 0.07 |  | 0.54 (0.11-2.61) | 0.44 |  | 0.29 (0.07-1.21) | 0.09 |
|  | Temperature (average over measuring period) | <5°C | 0.91 (0.68-1.23) | 0.55 |  | 0.91 (0.67-1.24) | 0.54 |  |  |  |
|  |  | 5-10°C | 0.86 (0.68-1.10) | 0.24 |  | 0.88 (0.69-1.14) | 0.35 |  |  |  |
|  |  | 15-20°C | 1.15 (0.90-1.47) | 0.28 |  | 1.11 (0.87-1.42) | 0.42 |  |  |  |
|  |  | 20-25°C | 1.22 (0.83-1.80) | 0.31 |  | 1.16 (0.79-1.71) | 0.45 |  |  |  |
|  |  | >25°C | 1.39 (0.64-3.03) | 0.40 |  | 1.33 (0.61-2.86) | 0.47 |  |  |  |
|  | Job status | (employed) | 0.62 (0.52-0.75) | <0.01 |  | 0.76 (0.59-0.99) | 0.04 |  | 0.77 (0.6-1.00) | 0.05 |
|  | Workdays | (days per week) | 0.90 (0.86-0.94) | <0.01 |  | 0.95 (0.89-1.01) | 0.11 |  | 0.95 (0.90-1.01) | 0.13 |
|  | Outdoors occupation | (yes) | 0.89 (0.64-1.24) | 0.50 |  | 0.99 (0.70-1.40) | 0.95 |  |  |  |
|  | COPD | (yes) | 0.91 (0.67-1.24) | 0.54 |  | 0.84 (0.61-1.16) | 0.29 |  |  |  |
|  | Asthma | (doctor diagnosed) | 0.99 (0.98-1.00) | 0.05 |  | 1.00 (0.99-1.01) | 0.52 |  |  |  |
|  | Hayfever | (self-reported) | 1.17 (0.96-1.43) | 0.12 |  | 1.18 (0.97-1.45) | 0.10 |  | 1.17 (0.96-1.43) | 0.11 |
|  | Betablocker usage | (yes) | 1.03 (0.76-1.39) | 0.85 |  | 0.88 (0.64-1.21) | 0.42 |  |  |  |
|  | History of heart diseases | (yes) | 1.24 (0.74-2.08) | 0.40 |  | 1.18 (0.69-2.01) | 0.54 |  |  |  |
|  | Person thinks health complaints are due to nearby livestock | (yes) | 1.00 (1.00-1.00) | 0.24 |  | 1.00 (1.00-1.00) | 0.40 |  |  |  |
|  | Time spent outdoors close to home | (hours per week) | 1.01 (1.00-1.02) | 0.24 |  | 1.00 (0.98-1.01) | 0.67 |  |  |  |
|  | Animal ownership | Dog | 1.00 (0.81-1.24) | 0.99 |  | 1.12 (0.90-1.40) | 0.30 |  |  |  |
|  |  | Livestock | 0.99 (0.98-1.00) | 0.06 |  | 1.00 (0.98-1.01) | 0.60 |  |  |  |
|  | | | | | | | | | | |
| Percentage of time spent in motorised  transport | Age | 45-55y | 1.13 (0.84-1.53) | 0.40 |  | 1.18 (0.87-1.61) | 0.28 |  | 1.19 (0.88-1.60) | 0.25 |
|  |  | 55-65y | 0.76 (0.58-1.01) | 0.06 |  | 0.91 (0.67-1.24) | 0.56 |  | 0.93 (0.69-1.25) | 0.63 |
|  |  | >65y | 0.63 (0.47-0.85) | <0.01 |  | 0.88 (0.61-1.27) | 0.50 |  | 0.88 (0.63-1.25) | 0.49 |
|  | Gender | Female | 0.97 (0.81-1.15) | 0.70 |  | 0.98 (0.81-1.18) | 0.81 |  | 0.96 (0.80-1.15) | 0.66 |
|  | BMI | Overweight  (25-30 kg/m2) | 1.06 (0.87-1.30) | 0.55 |  | 1.10 (0.90-1.34) | 0.36 |  |  |  |
|  |  | Obese  (>30 kg/m2) | 0.96 (0.75-1.24) | 0.77 |  | 1.01 (0.78-1.31) | 0.93 |  |  |  |
|  | Smoker | Former | 0.88 (0.73-1.05) | 0.16 |  | 1.02 (0.84-1.23) | 0.85 |  |  |  |
|  |  | Current | 0.96 (0.69-1.35) | 0.83 |  | 1.02 (0.73-1.43) | 0.89 |  |  |  |
|  | Education level | Medium | 1.42 (1.14-1.76) | <0.01 |  | 1.31 (1.04-1.64) | 0.02 |  | 1.29 (1.03-1.60) | 0.02 |
|  |  | High | 1.51 (1.19-1.91) | <0.01 |  | 1.40 (1.09-1.81) | 0.01 |  | 1.37 (1.08-1.74) | 0.01 |
|  | Duration of rainfall | (% time over measuring period) | 0.81 (0.20-3.34) | 0.77 |  | 1.02 (0.21-4.90) | 0.98 |  |  |  |
|  | Temperature (average over measuring period) | <5°C | 0.74 (0.55-0.99) | 0.04 |  | 0.77 (0.57-1.05) | 0.09 |  |  |  |
|  |  | 5-10°C | 0.96 (0.75-1.22) | 0.73 |  | 0.96 (0.74-1.23) | 0.72 |  |  |  |
|  |  | 15-20°C | 0.98 (0.77-1.25) | 0.89 |  | 0.97 (0.76-1.24) | 0.82 |  |  |  |
|  |  | 20-25°C | 0.91 (0.62-1.34) | 0.65 |  | 0.96 (0.66-1.41) | 0.85 |  |  |  |
|  |  | >25°C | 1.50 (0.70-3.21) | 0.30 |  | 1.66 (0.78-3.56) | 0.19 |  |  |  |
|  | Job status | (employed) | 1.52 (1.26-1.82) | <0.01 |  | 1.04 (0.80-1.34) | 0.77 |  |  |  |
|  | Workdays | (days per week) | 1.12 (1.07-1.17) | <0.01 |  | 1.06 (1.00-1.13) | 0.05 |  | 1.08 (1.03-1.13) | <0.01 |
|  | Outdoors occupation | (yes) | 1.35 (0.98-1.87) | 0.07 |  | 1.24 (0.88-1.74) | 0.23 |  |  |  |
|  | COPD | (yes) | 0.93 (0.68-1.26) | 0.63 |  | 1.02 (0.74-1.40) | 0.91 |  |  |  |
|  | Asthma | (doctor diagnosed) | 1.00 (0.99-1.01) | 0.86 |  | 1.00 (0.99-1.01) | 0.89 |  |  |  |
|  | Hayfever | (self-reported) | 0.96 (0.78-1.17) | 0.65 |  | 0.94 (0.77-1.15) | 0.54 |  |  |  |
|  | Betablocker usage | (yes) | 0.91 (0.68-1.22) | 0.52 |  | 1.06 (0.78-1.45) | 0.71 |  |  |  |
|  | History of heart diseases | (yes) | 1.34 (0.81-2.22) | 0.25 |  | 1.66 (0.98-2.81) | 0.06 |  | 1.67 (1.01-2.75) | 0.05 |
|  | Person thinks health complaints are due to nearby livestock | (yes) | 1.00 (1.00-1.00) | 0.75 |  | 1.00 (1.00-1.00) | 0.52 |  |  |  |
|  | Time spent outdoors close to home | (hours per week) | 0.99 (0.98-1.00) | 0.10 |  | 0.99 (0.98-1.01) | 0.35 |  |  |  |
|  | Animal ownership | Dog | 1.34 (1.09-1.64) | 0.01 |  | 1.27 (1.02-1.57) | 0.03 |  | 1.25 (1.02-1.54) | 0.04 |
|  |  | Livestock | 1.01 (1.00-1.02) | 0.29 |  | 1.01 (1.00-1.02) | 0.17 |  | 1.01 (1.00-1.02) | 0.16 |

**Supp. Table 2** overview of final linear models for percentages of time (spent: outdoors, in non-motorised and motorised transport), univariate models, full models and supervised stepwise backwards selection (SSBS) models. Green boxes indicate statistical significant outcomes, yellow boxes indicate borderline significant outcomes. **Bold font** for the explanatory factors indicates that they are (borderline-) significant for all three modelling approaches.

7. Supplementary Table 3 (distances from home address)

| **Supp. Table 3** Overview model variables  (distances from home address) | | | **Univariate models** | | | **Full models** | | | **SSBS models** | | |
| --- | --- | --- | --- | --- | --- | --- | --- | --- | --- | --- | --- |
| **Outcome** | **Variable** | **Category** | **GMR (95% CI)** | **P.value** |  | **GMR (95% CI)** | **P.value** |  | **GMR (95% CI)** | **P.value** | |
| Average distances from home while walking | Age | 45-55y | 0.83 (0.56-1.23) | 0.35 |  | 0.82 (0.55-1.22) | 0.33 |  | 0.88 (0.59-1.30) | 0.51 | |
|  |  | 55-65y | 0.64 (0.44-0.93) | 0.02 |  | 0.72 (0.48-1.07) | 0.11 |  | 0.76 (0.52-1.12) | 0.17 | |
|  |  | >65y | 0.50 (0.34-0.73) | <0.01 |  | 0.61 (0.38-0.99) | 0.05 |  | 0.65 (0.41-1.02) | 0.06 | |
|  | Gender | Female | 1.14 (0.90-1.44) | 0.28 |  | 1.06 (0.83-1.36) | 0.64 |  | 1.07 (0.84-1.36) | 0.57 | |
|  | BMI | Overweight  (25-30 kg/m2) | 1.06 (0.82-1.38) | 0.65 |  | 1.18 (0.91-1.53) | 0.22 |  |  |  | |
|  |  | Obese  (>30 kg/m2) | 0.85 (0.60-1.18) | 0.33 |  | 0.99 (0.70-1.38) | 0.94 |  |  |  | |
|  | Smoker | Former | 0.87 (0.68-1.11) | 0.25 |  | 1.03 (0.80-1.33) | 0.79 |  |  |  | |
|  |  | Current | 0.90 (0.58-1.41) | 0.65 |  | 1.07 (0.69-1.66) | 0.77 |  |  |  | |
|  | **Education level** | Medium | 1.52 (1.14-2.02) | <0.01 |  | 1.31 (0.97-1.76) | 0.08 |  | 1.31 (0.98-1.76) | 0.06 | |
|  |  | **High** | 1.93 (1.42-2.63) | <0.01 |  | 1.54 (1.11-2.15) | 0.01 |  | 1.55 (1.13-2.14) | 0.01 | |
|  | Duration of rainfall | (% time over measuring period) | 0.21 (0.03-1.35) | 0.10 |  | 0.36 (0.05-2.80) | 0.33 |  | 0.18 (0.03-1.12) | 0.07 | |
|  | Temperature (average over measuring period) | <5°C | 0.76 (0.52-1.13) | 0.18 |  | 0.77 (0.52-1.14) | 0.19 |  |  |  | |
|  |  | 5-10°C | 0.87 (0.63-1.19) | 0.37 |  | 0.93 (0.67-1.29) | 0.65 |  |  |  | |
|  |  | 15-20°C | 1.25 (0.91-1.73) | 0.17 |  | 1.30 (0.95-1.79) | 0.10 |  |  |  | |
|  |  | 20-25°C | 0.79 (0.47-1.31) | 0.35 |  | 0.85 (0.51-1.40) | 0.52 |  |  |  | |
|  |  | >25°C | 1.12 (0.41-3.08) | 0.82 |  | 1.06 (0.39-2.88) | 0.91 |  |  |  | |
|  | Job status | (employed) | 1.41 (1.10-1.80) | 0.01 |  | 0.93 (0.66-1.30) | 0.66 |  |  |  | |
|  | **Workdays** | **(days per week)** | 1.11 (1.05-1.17) | <0.01 |  | 1.08 (1.00-1.17) | 0.05 |  | 1.07 (1.00-1.15) | 0.04 | |
|  | Outdoors occupation | (yes) | 0.72 (0.47-1.10) | 0.12 |  | 0.70 (0.45-1.10) | 0.12 |  | 0.72 (0.46-1.12) | 0.14 | |
|  | COPD | (yes) | 0.86 (0.57-1.29) | 0.46 |  | 0.98 (0.65-1.48) | 0.93 |  |  |  | |
|  | Asthma | (doctor diagnosed) | 0.99 (0.98-1.00) | 0.14 |  | 0.99 (0.98-1.01) | 0.30 |  |  |  | |
|  | Hayfever | (self-reported) | 1.33 (1.02-1.73) | 0.03 |  | 1.19 (0.92-1.55) | 0.18 |  | 1.21 (0.93-1.56) | 0.15 | |
|  | Betablocker usage | (yes) | 0.60 (0.41-0.88) | 0.01 |  | 0.71 (0.47-1.06) | 0.10 |  | 0.68 (0.46-1.01) | 0.05 | |
|  | History of heart diseases | (yes) | 0.68 (0.35-1.33) | 0.26 |  | 0.85 (0.42-1.69) | 0.63 |  |  |  | |
|  | Person thinks health complaints are due to nearby livestock | (yes) | 1.00 (1.00-1.00) | 0.86 |  | 1.00 (1.00-1.00) | 0.42 |  |  |  | |
|  | Time spent outdoors close to home | (hours per week) | 0.98 (0.96-0.99) | 0.01 |  | 0.99 (0.97-1.01) | 0.32 |  |  |  | |
|  | **Animal ownership** | **Dog** | 0.58 (0.44-0.76) | <0.01 |  | 0.51 (0.39-0.68) | <0.01 |  | 0.51 (0.39-0.67) | <0.01 | |
|  |  | Livestock | 1.00 (0.99-1.02) | 0.61 |  | 1.01 (1.00-1.03) | 0.16 |  |  |  | |
|  | | | | | | | | | | | |
| Average distances from home while biking | Age | 45-55y | 1.05 (0.76-1.46) | 0.76 |  | 1.01 (0.72-1.42) | 0.94 |  | 1.07 (0.76-1.48) | 0.71 | |
|  |  | 55-65y | 1.08 (0.80-1.47) | 0.61 |  | 0.98 (0.70-1.38) | 0.91 |  | 1.11 (0.81-1.53) | 0.52 | |
|  |  | >65y | 0.94 (0.68-1.29) | 0.69 |  | 0.8 (0.54-1.20) | 0.29 |  | 0.96 (0.68-1.35) | 0.81 | |
|  | Gender | Female | 0.93 (0.77-1.13) | 0.45 |  | 0.96 (0.78-1.18) | 0.70 |  | 0.95 (0.78-1.15) | 0.59 | |
|  | BMI | Overweight (25-30 kg/m2) | 1.10 (0.89-1.37) | 0.38 |  | 1.12 (0.90-1.40) | 0.30 |  |  |  | |
|  |  | Obese  (>30 kg/m2) | 0.91 (0.69-1.20) | 0.52 |  | 0.99 (0.75-1.32) | 0.97 |  |  |  | |
|  | Smoker | Former | 1.09 (0.89-1.34) | 0.38 |  | 1.13 (0.91-1.40) | 0.26 |  |  |  | |
|  |  | Current | 0.77 (0.53-1.10) | 0.15 |  | 0.81 (0.56-1.17) | 0.26 |  |  |  | |
|  | Education level | Medium | 1.04 (0.82-1.32) | 0.72 |  | 1.05 (0.82-1.35) | 0.70 |  | 1.03 (0.80-1.31) | 0.84 | |
|  |  | High | 1.26 (0.97-1.63) | 0.08 |  | 1.22 (0.92-1.61) | 0.16 |  | 1.20 (0.92-1.57) | 0.17 | |
|  | Duration of rainfall | (% time over measuring period) | 0.53 (0.11-2.49) | 0.42 |  | 1.15 (0.20-6.53) | 0.87 |  |  |  | |
|  | Temperature (average over measuring period) | <5°C | 0.74 (0.53-1.02) | 0.06 |  | 0.73 (0.52-1.02) | 0.07 |  |  |  | |
|  |  | 5-10°C | 0.92 (0.71-1.20) | 0.54 |  | 0.91 (0.69-1.21) | 0.53 |  |  |  | |
|  |  | 15-20°C | 1.05 (0.80-1.37) | 0.74 |  | 1.06 (0.81-1.39) | 0.67 |  |  |  | |
|  |  | 20-25°C | 1.36 (0.89-2.07) | 0.15 |  | 1.37 (0.90-2.1) | 0.14 |  |  |  | |
|  |  | >25°C | 1.07 (0.47-2.47) | 0.87 |  | 1.06 (0.46-2.46) | 0.90 |  |  |  | |
|  | Job status | (employed) | 0.99 (0.80-1.21) | 0.90 |  | 0.86 (0.64-1.14) | 0.29 |  |  |  | |
|  | Workdays | (days per week) | 1.02 (0.97-1.06) | 0.52 |  | 1.01 (0.94-1.08) | 0.86 |  |  |  | |
|  | Outdoors occupation | (yes) | 1.00 (0.70-1.43) | 0.98 |  | 1.03 (0.70-1.51) | 0.87 |  |  |  | |
|  | COPD | (yes) | 1.02 (0.73-1.43) | 0.90 |  | 1.06 (0.75-1.50) | 0.74 |  |  |  | |
|  | Asthma | (doctor diagnosed) | 0.99 (0.99-1.00) | 0.21 |  | 1.00 (0.99-1.01) | 0.38 |  |  |  | |
|  | Hayfever | (self-reported) | 1.25 (1.01-1.56) | 0.04 |  | 1.22 (0.98-1.51) | 0.08 |  | 1.23 (0.99-1.53) | 0.06 | |
|  | **Betablocker usage** | **(yes)** | 0.62 (0.45-0.85) | <0.01 |  | 0.60 (0.43-0.85) | <0.01 |  | 0.63 (0.45-0.88) | 0.01 | |
|  | History of heart diseases | (yes) | 1.00 (0.58-1.74) | 1.00 |  | 1.27 (0.71-2.28) | 0.42 |  |  |  | |
|  | Person thinks health complaints are due to nearby livestock | (yes) | 1.00 (1.00-1.00) | 0.12 |  | 1.00 (1.00-1.00) | 0.15 |  | 1.00 (1.00-1.01) | 0.07 | |
|  | Time spent outdoors close to home | (hours per week) | 0.99 (0.98-1.01) | 0.33 |  | 1.00 (0.98-1.01) | 0.54 |  |  |  | |
|  | **Animal ownership** | **Dog** | 0.76 (0.60-0.95) | 0.02 |  | 0.73 (0.58-0.93) | 0.01 |  | 0.73 (0.58-0.92) | 0.01 | |
|  |  | Livestock | 0.99 (0.98-1.01) | 0.33 |  | 0.99 (0.98-1.01) | 0.46 |  |  |  | |
|  | | | | | | | | | | | |
| Average distances from home while in motorised transport | Age | 45-55y | 0.92 (0.65-1.30) | 0.63 |  | 0.90 (0.63-1.28) | 0.55 |  | 0.89 (0.63-1.26) | | 0.52 |
|  |  | 55-65y | 0.87 (0.63-1.19) | 0.38 |  | 0.91 (0.64-1.30) | 0.60 |  | 0.90 (0.64-1.26) | | 0.53 |
|  |  | >65y | 0.70 (0.50-0.98) | 0.04 |  | 0.82 (0.54-1.25) | 0.36 |  | 0.81 (0.54-1.2) | | 0.29 |
|  | Gender | Female | 0.83 (0.67-1.01) | 0.06 |  | 0.85 (0.68-1.05) | 0.13 |  | 0.88 (0.71-1.08) | | 0.21 |
|  | BMI | Overweight (25-30 kg/m2) | 1.01 (0.80-1.27) | 0.92 |  | 1.01 (0.80-1.27) | 0.97 |  |  | |  |
|  |  | Obese  (>30 kg/m2) | 1.12 (0.84-1.51) | 0.44 |  | 1.14 (0.85-1.54) | 0.37 |  |  | |  |
|  | Smoker | Former | 0.97 (0.78-1.20) | 0.78 |  | 1.06 (0.85-1.32) | 0.60 |  |  | |  |
|  |  | Current | 1.10 (0.75-1.62) | 0.63 |  | 1.23 (0.84-1.81) | 0.29 |  |  | |  |
|  | **Education level** | Medium | 1.02 (0.80-1.31) | 0.85 |  | 0.96 (0.73-1.24) | 0.73 |  | 0.95 (0.74-1.23) | | 0.72 |
|  |  | **High** | 1.58 (1.20-2.07) | <0.01 |  | 1.40 (1.05-1.88) | 0.02 |  | 1.40 (1.06-1.85) | | 0.02 |
|  | Duration of rainfall | (% time over measuring period) | 2.10 (0.41-10.82) | 0.37 |  | 1.11 (0.18-6.78) | 0.91 |  |  | |  |
|  | **Temperature (average over measuring period)** | <5°C | 0.87 (0.62-1.22) | 0.41 |  | 0.83 (0.59-1.18) | 0.30 |  | 0.86 (0.61-1.20) | | 0.37 |
|  |  | 5-10°C | 1.02 (0.77-1.34) | 0.89 |  | 0.99 (0.74-1.32) | 0.92 |  | 0.98 (0.75-1.29) | | 0.91 |
|  |  | 15-20°C | 0.95 (0.72-1.26) | 0.72 |  | 0.95 (0.72-1.26) | 0.74 |  | 0.94 (0.71-1.25) | | 0.68 |
|  |  | **20-25°**C | 0.54 (0.35-0.84) | 0.01 |  | 0.49 (0.32-0.77) | <0.01 |  | 0.50 (0.32-0.78) | | <0.01 |
|  |  | >25°C | 0.63 (0.26-1.52) | 0.31 |  | 0.55 (0.23-1.33) | 0.18 |  | 0.56 (0.24-1.34) | | 0.19 |
|  | Job status | (employed) | 1.39 (1.12-1.72) | <0.01 |  | 1.13 (0.84-1.52) | 0.42 |  |  | |  |
|  | Workdays | (days per week) | 1.09 (1.04-1.14) | <0.01 |  | 1.06 (0.98-1.13) | 0.13 |  | 1.07 (1.01-1.13) | | 0.02 |
|  | Outdoors occupation | (yes) | 1.05 (0.72-1.52) | 0.80 |  | 0.93 (0.63-1.39) | 0.73 |  |  | |  |
|  | COPD | (yes) | 1.43 (1.01-2.04) | 0.05 |  | 1.42 (0.99-2.04) | 0.06 |  | 1.51 (1.06-2.15) | | 0.02 |
|  | **Asthma** | **(doctor diagnosed)** | 0.99 (0.98-1.00) | 0.04 |  | 0.99 (0.98-1.00) | 0.02 |  | 0.99 (0.98-1.00) | | 0.01 |
|  | Hayfever | (self-reported) | 1.11 (0.88-1.39) | 0.38 |  | 1.13 (0.90-1.42) | 0.29 |  |  | |  |
|  | Betablocker usage | (yes) | 0.92 (0.66-1.29) | 0.63 |  | 1.11 (0.77-1.60) | 0.56 |  |  | |  |
|  | History of heart diseases | (yes) | 0.73 (0.41-1.30) | 0.28 |  | 0.71 (0.39-1.31) | 0.27 |  |  | |  |
|  | Person thinks health complaints are due to nearby livestock | (yes) | 1.00 (1.00-1.01) | 0.07 |  | 1.00 (1.00-1.01) | 0.01 |  | 1.00 (1.00-1.01) | | 0.02 |
|  | Time spent outdoors close to home | (hours per week) | 0.99 (0.97-1.00) | 0.08 |  | 0.99 (0.98-1.01) | 0.49 |  |  | |  |
|  | Animal ownership | Dog | 0.90 (0.71-1.15) | 0.41 |  | 0.90 (0.70-1.15) | 0.40 |  |  | |  |
|  |  | Livestock | 1.01 (1.00-1.02) | 0.21 |  | 1.01 (0.99-1.03) | 0.20 |  | 1.01 (1.00-1.02) | | 0.05 |

**Supp. Table 3** overview of final linear models for average distances from home (while: walking, biking, motorised), univariate models, full models and supervised stepwise backwards selection (SSBS) models. Green boxes indicate statistical significant outcomes, yellow boxes indicate borderline significant outcomes. **Bold font** for the explanatory factors indicates that they are (borderline-) significant for all three modelling approaches.

***Sensitivity analyses***

***8.*** Buffer sizes around the home address, 60m buffer versus 20m buffer.

| 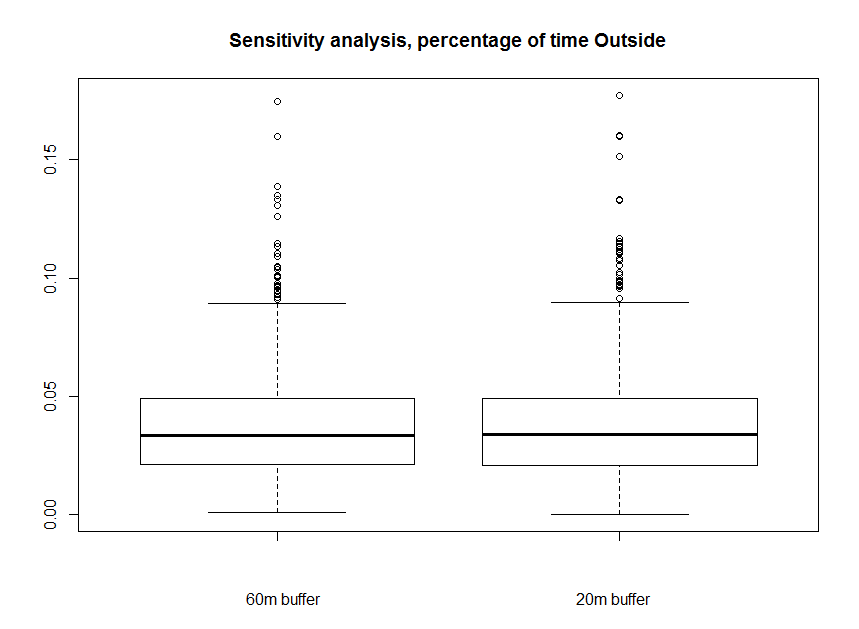 | 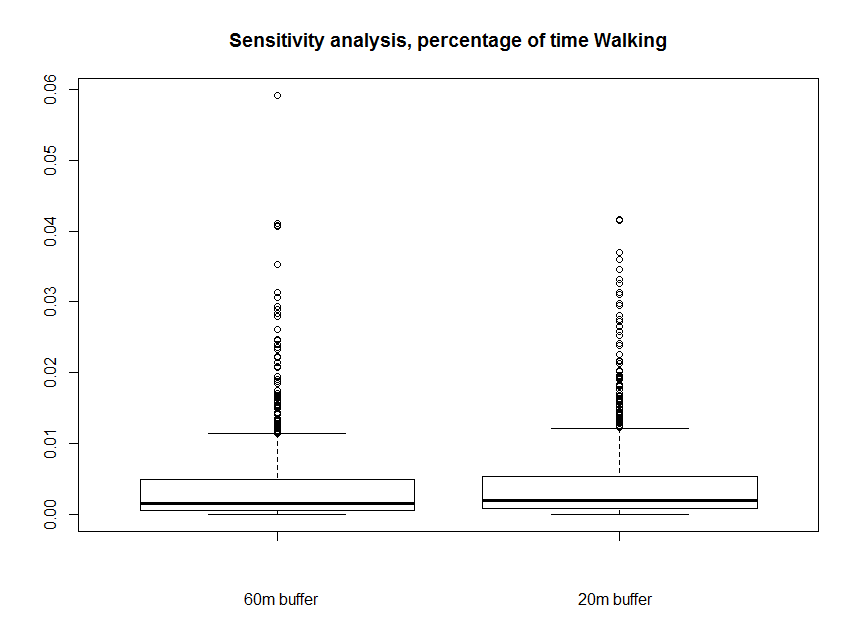 |
| --- | --- |
| 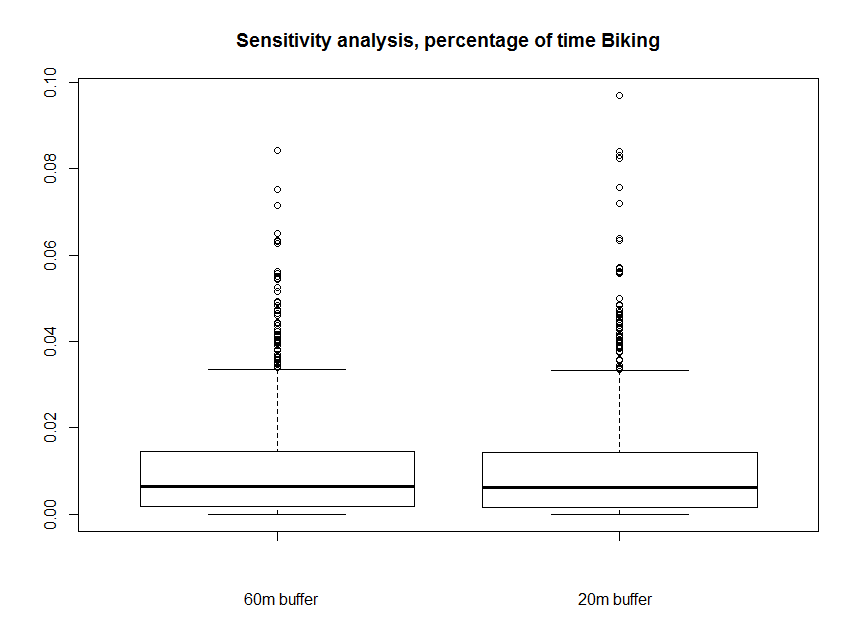 | 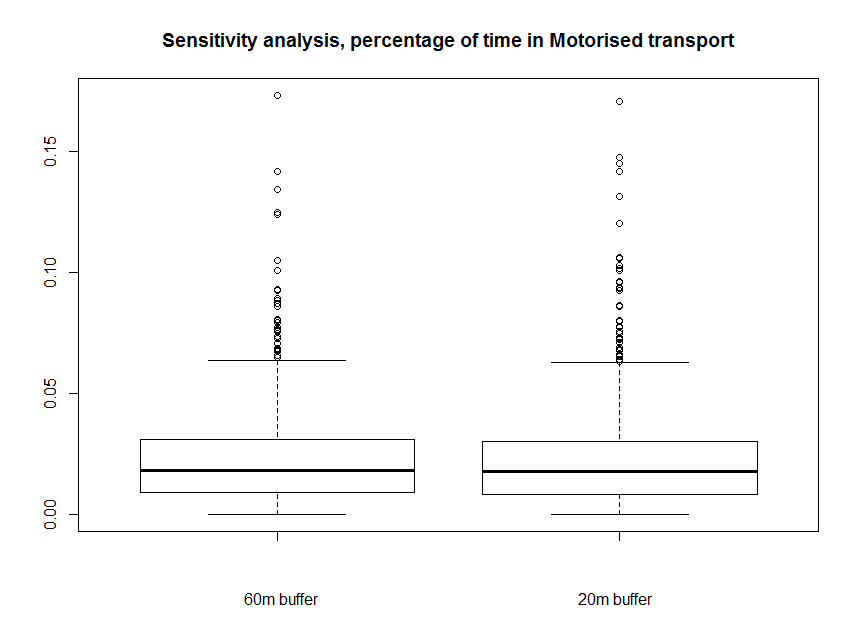 |

**Supp. Figure 6** Boxplots, GPS data is used to compare the influence of buffer sizes on percentages of time spent: A. outside, B. walking, C. biking, D. in motorised transport, after assignment of indoor/outdoor and to the specific transport modes.

| **Outcome** | **Mean of the difference (95% CI)** | **T-value** | **P-value** |
| --- | --- | --- | --- |
| **Percentage of time Outside** | -3.33*10^-4^  (-2.49*10^-3^, 1.82*10^-3^) | -0.30 | 0.76 |
| **Percentage of time Walking** | -4.02*10^-4^  (-9.44*10^-4^, 1.40*10^-4^) | -1.46 | 0.15 |
| **Percentage of time Biking** | -1.53*10^-4^  (-1.23*10^-3^,9.24*10^-4^) | -0.28 | 0.78 |
| **Percentage of time Motorised** | 2.39*10^-4^  (-1.58*10^-3^, 2.06*10^-3^) | 0.26 | 0.80 |

**Supp. Table 4** Overview of T-test outcomes for the comparison of buffer sizes on percentages of time spent: outside, walking, biking, in motorised transport, after assignment of indoor/outdoor and to the specific transport modes. No statistical significant differences in percentages of time spent were identified between the two buffer sizes, therefore we decided to work with the previously assigned 60m buffers for all analyses.

| **Supp. Table 5** Sensitivity analyses SSBS models  9. Supplementary Table 5 sensitivity analysis SSBS models (percentages of time) full dataset and dataset without people deviating from normal weekly mobility pattern  Full dataset versus dataset people reporting a ‘normal week’ (percentages of time) | | | **SSBS models (Full dataset N=870)** | | | **SSBS models, only if not deviated from normal week (N=635)** | |
| --- | --- | --- | --- | --- | --- | --- | --- |
| **Outcome** | **Variable** | **Category** | **GMr (95% CI)** | **P-value** |  | **GMr (95% CI)** | **P.value** |
| *Percentage of time*  *spent outside* | Age | 45-55y | 1.12 (0.94-1.34) | 0.21 |  | 1.15 (0.93-1.43) | 0.20 |
|  |  | 55-65y | 1.07 (0.89-1.28) | 0.46 |  | 1.07 (0.86-1.33) | 0.54 |
|  |  | >65y | 1.02 (0.83-1.26) | 0.84 |  | 1.19 (0.92-1.54) | 0.19 |
|  | Gender | Female | 0.86 (0.77-0.95) | <0.01 |  | 0.86 (0.75-0.98) | 0.02 |
|  | Education level | Medium | 1.07 (0.94-1.22) | 0.31 |  | 1.09 (0.93-1.28) | 0.28 |
|  |  | High | 1.14 (0.98-1.31) | 0.09 |  | 1.17 (0.98-1.40) | 0.08 |
|  | Temperature (average over measuring period) | <5°C | 0.80 (0.67-0.95) | 0.01 |  | 0.80 (0.64-0.99) | 0.04 |
|  |  | 5-10°C | 1.00 (0.86-1.15) | 0.95 |  | 0.96 (0.81-1.14) | 0.66 |
|  |  | 15-20°C | 0.98 (0.84-1.13) | 0.74 |  | 0.97 (0.82-1.15) | 0.74 |
|  |  | 20-25°C | 0.94 (0.75-1.17) | 0.56 |  | 0.84 (0.63-1.13) | 0.25 |
|  |  | >25°C | 1.40 (0.89-2.19) | 0.14 |  | 1.58 (0.89-2.78) | 0.12 |
|  | Workdays | (days per week) | 1.02 (0.99-1.05) | 0.17 |  | 1.03 (1.00-1.07) | 0.06 |
|  | Asthma | (doctor diagnosed) | 1.00 (0.99-1.00) | 0.13 |  | 1.00 (0.99-1.00) | 0.12 |
|  | History of heart diseases | (yes) | 1.28 (0.94-1.73) | 0.11 |  | 1.07 (0.75-1.51) | 0.72 |
|  | Animal ownership | Dog | 1.15 (1.02-1.31) | 0.03 |  | 1.22 (1.05-1.42) | 0.01 |
|  |  | Livestock | 1.00 (1.00-1.01) | 0.15 |  | 1.00 (1.00-1.01) | 0.37 |
| *Percentage of Time*  *spent in non-motorised*  *transport* | Age | 45-55y | 1.25 (0.92-1.70) | 0.15 |  | 1.29 (0.91-1.83) | 0.15 |
|  |  | 55-65y | 1.43 (1.06-1.95) | 0.02 |  | 1.32 (0.92-1.88) | 0.13 |
|  |  | >65y | 1.38 (0.97-1.97) | 0.07 |  | 1.46 (0.96-2.21) | 0.08 |
|  | Gender | Female | 0.99 (0.82-1.19) | 0.9 |  | 0.98 (0.79-1.21) | 0.82 |
|  | BMI | Overweight (25-30 kg/m2) | 0.96 (0.78-1.17) | 0.66 |  | 1.03 (0.82-1.30) | 0.81 |
|  |  | Obese  (>30 kg/m2) | 0.69 (0.54-0.90) | 0.01 |  | 0.81 (0.60-1.09) | 0.16 |
|  | Smoker | Former | 0.93 (0.77-1.13) | 0.49 |  | 0.95 (0.76-1.19) | 0.65 |
|  |  | Current | 0.64 (0.46-0.89) | 0.01 |  | 0.60 (0.41-0.86) | 0.01 |
|  | Education level | Medium | 0.95 (0.76-1.20) | 0.68 |  | 0.99 (0.77-1.29) | 0.96 |
|  |  | High | 0.90 (0.70-1.16) | 0.42 |  | 0.92 (0.68-1.23) | 0.57 |
|  | Duration of rainfall | (% time over measuring period) | 0.29 (0.07-1.21) | 0.09 |  | 0.38 (0.07-2.01) | 0.25 |
|  | Job status | (employed) | 0.77 (0.60-1.00) | 0.05 |  | 0.83 (0.61-1.13) | 0.23 |
|  | Workdays | (days per week) | 0.95 (0.90-1.01) | 0.13 |  | 0.92 (0.86-0.99) | 0.02 |
|  | Hayfever | (self-reported) | 1.17 (0.96-1.43) | 0.11 |  | 1.07 (0.85-1.35) | 0.54 |
| *Percentage of Time*  *spent in motorised*  *transport* | Age | 45-55y | 1.19 (0.88-1.60) | 0.25 |  | 1.25 (0.89-1.77) | 0.20 |
|  |  | 55-65y | 0.93 (0.69-1.25) | 0.63 |  | 1.06 (0.75-1.49) | 0.73 |
|  |  | >65y | 0.88 (0.63-1.25) | 0.49 |  | 0.99 (0.65-1.49) | 0.95 |
|  | Gender | Female | 0.96 (0.80-1.15) | 0.66 |  | 0.92 (0.74-1.13) | 0.42 |
|  | Education level | Medium | 1.29 (1.03-1.60) | 0.02 |  | 1.34 (1.04-1.73) | 0.02 |
|  |  | High | 1.37 (1.08-1.74) | 0.01 |  | 1.42 (1.07-1.89) | 0.02 |
|  | Workdays | (days per week) | 1.08 (1.03-1.13) | <0.01 |  | 1.11 (1.05-1.17) | <0.01 |
|  | History of heart diseases | (yes) | 1.67 (1.01-2.75) | 0.05 |  | 1.44 (0.82-2.51) | 0.20 |
|  | Animal ownership | Dog | 1.25 (1.02-1.54) | 0.04 |  | 1.35 (1.06-1.72) | 0.01 |
|  |  | Livestock | 1.01 (1.00-1.02) | 0.16 |  | 1.01 (0.99-1.02) | 0.37 |

**Supp. Table 5** Sensitivity analyses for percentages of time (spent: outdoors, in non-motorised and motorised transport) for people indicating to have had a ‘normal week’. In questionnaire 2 (Q2), regarding study adherence, we inquired whether people had had a ‘normal week’. Of our participants 73% indicated to have had a ‘normal week’, we reanalysed our supervised stepwise backwards selection (SSBS) models with this subpopulation and overall found no material effects on our estimates.

| **10. Table 6** Sensitivity analyses SSBS models  10. Supplementary Table 6 sensitivity analysis SSBS models (distances from home address) full dataset and dataset without people deviating from normal weekly mobility pattern  Full dataset versus dataset people reporting a ‘normal week’ (distances from home address) | | | **SSBS models (Full dataset N=870)** | | | **SSBS models, only if not deviated from normal week (N=635)** | |
| --- | --- | --- | --- | --- | --- | --- | --- |
| **Outcome** | **Variable** | **Category** | **GMr (95% CI)** | **P-value** |  | **GMr (95% CI)** | **P.value** |
| *Percentage of time*  *spent outside* | Age | 45-55y | 0.88 (0.59-1.30) | 0.51 |  | 0.88 (0.53-1.46) | 0.62 |
|  |  | 55-65y | 0.76 (0.52-1.12) | 0.17 |  | 0.75 (0.45-1.25) | 0.27 |
|  |  | >65y | 0.65 (0.41-1.02) | 0.06 |  | 0.84 (0.46-1.55) | 0.58 |
|  | Gender | Female | 1.07 (0.84-1.36) | 0.57 |  | 1.18 (0.86-1.63) | 0.31 |
|  | Education level | Medium | 1.31 (0.98-1.76) | 0.06 |  | 1.35 (0.92-1.96) | 0.12 |
|  |  | High | 1.55 (1.13-2.14) | 0.01 |  | 1.82 (1.18-2.80) | 0.01 |
|  | Duration of rainfall | (% time over measuring period) | 0.18 (0.03-1.12) | 0.07 |  | 1.55 (0.14-17.48) | 0.72 |
|  | Workdays | (days per week) | 1.07 (1.00-1.15) | 0.04 |  | 1.12 (1.02-1.22) | 0.02 |
|  | Outdoors occupation | (yes) | 0.72 (0.46-1.12) | 0.14 |  | 0.98 (0.56-1.71) | 0.93 |
|  | Hayfever | (self-reported) | 1.21 (0.93-1.56) | 0.15 |  | 1.27 (0.90-1.77) | 0.17 |
|  | Betablocker usage | (yes) | 0.68 (0.46-1.01) | 0.05 |  | 0.77 (0.46-1.30) | 0.33 |
|  | Animal ownership | Dog | 0.51 (0.39-0.67) | <0.01 |  | 0.51 (0.35-0.72) | <0.01 |
| *Percentage of Time*  *spent in non-motorised*  *transport* | Age | 45-55y | 1.07 (0.76-1.48) | 0.71 |  | 1.13 (0.77-1.65) | 0.54 |
|  |  | 55-65y | 1.11 (0.81-1.53) | 0.52 |  | 1.04 (0.72-1.50) | 0.83 |
|  |  | >65y | 0.96 (0.68-1.35) | 0.81 |  | 1.04 (0.69-1.56) | 0.86 |
|  | Gender | Female | 0.95 (0.78-1.15) | 0.59 |  | 0.86 (0.68-1.08) | 0.20 |
|  | Education level | Medium | 1.03 (0.80-1.31) | 0.84 |  | 0.94 (0.71-1.24) | 0.66 |
|  |  | High | 1.20 (0.92-1.57) | 0.17 |  | 1.08 (0.79-1.48) | 0.61 |
|  | Hayfever | (self-reported) | 1.23 (0.99-1.53) | 0.06 |  | 1.25 (0.97-1.61) | 0.08 |
|  | Betablocker usage | (yes) | 0.63 (0.45-0.88) | 0.01 |  | 0.62 (0.42-0.90) | 0.01 |
|  | Person thinks health complaints are due to nearby livestock | (yes) | 1.00 (1.00-1.01) | 0.07 |  | 1.00 (1.00-1.01) | 0.15 |
|  | Animal ownership | Dog | 0.73 (0.58-0.92) | 0.01 |  | 0.79 (0.61-1.03) | 0.08 |
| *Percentage of Time*  *spent in motorised*  *transport* | Age | 45-55y | 0.89 (0.63-1.26) | 0.52 |  | 0.96 (0.65-1.42) | 0.84 |
|  |  | 55-65y | 0.90 (0.64-1.26) | 0.53 |  | 0.92 (0.63-1.36) | 0.69 |
|  |  | >65y | 0.81 (0.54-1.20) | 0.29 |  | 1.06 (0.67-1.67) | 0.81 |
|  | Gender | Female | 0.88 (0.71-1.08) | 0.21 |  | 0.88 (0.69-1.12) | 0.29 |
|  | Education level | Medium | 0.95 (0.74-1.23) | 0.72 |  | 1.07 (0.80-1.43) | 0.64 |
|  |  | High | 1.40 (1.06-1.85) | 0.02 |  | 1.64 (1.19-2.25) | <0.01 |
|  | Temperature (average over measuring period) | <5°C | 0.86 (0.61-1.20) | 0.37 |  | 1.17 (0.78-1.74) | 0.45 |
|  |  | 5-10°C | 0.98 (0.75-1.29) | 0.91 |  | 1.03 (0.76-1.40) | 0.85 |
|  |  | 15-20°C | 0.94 (0.71-1.25) | 0.68 |  | 0.89 (0.65-1.21) | 0.46 |
|  |  | 20-25°C | 0.50 (0.32-0.78) | <0.01 |  | 0.56 (0.33-0.96) | 0.03 |
|  |  | >25°C | 0.56 (0.24-1.34) | 0.19 |  | 0.56 (0.20-1.57) | 0.27 |
|  | Workdays | (days per week) | 1.07 (1.01-1.13) | 0.02 |  | 1.11 (1.04-1.19) | <0.01 |
|  | COPD | (yes) | 1.51 (1.06-2.15) | 0.02 |  | 1.38 (0.93-2.05) | 0.11 |
|  | Asthma | (doctor diagnosed) | 0.99 (0.98-1.00) | 0.01 |  | 0.99 (0.98-1.00) | 0.01 |
|  | Person thinks health complaints are due to nearby livestock | (yes) | 1.00 (1.00-1.01) | 0.02 |  | 1.00 (1.00-1.01) | 0.29 |
|  | Animal ownership | Livestock | 1.01 (1.00-1.02) | 0.05 |  | 1.01 (1.00-1.03) | 0.10 |

**Supp. Table 6** Sensitivity analyses for average distances from home (while: walking, biking, motorised)for people indicating to have had a ‘normal week’. In questionnaire 2 (Q2), regarding study adherence, we inquired whether people had had a ‘normal week’. Of our participants 73% indicated to have had a ‘normal week’, we reanalysed our supervised stepwise backwards selection (SSBS) models with this subpopulation and overall found no material effects on our estimates with the possible exception of duration of rainfall.

***Questionnaires***

***11.*** *Questionnaire (Q1)*

Translated from Dutch to English, highlighted text indicates comment by GK.

VGO GPS study Questionnaire 1 (filled in **prior** to GPS carrying)

This questionnaire includes 10 questions, among which 8 multiple-choice questions.

Please indicate what is applicable to your situation by filling in the boxes (⚫).

X

If you make a mistake, please indicate this with a cross trough the mistake (⚫) 🡪 and afterwards fill in the right answer (⚫).

For some questions we ask you to estimate durations of specific travel modes, can you please estimate durations for a normal week and can you be as specific as possible?

General questions

1. What is the average amount of **hours per day** you spend outdoors?

**Weekdays** (Monday-Friday) **Weekend** (Saturday and Sunday)

_└────┘_hours _└────┘_hours

1. Are your currently **employed** (either a paid or an unpaid voluntary position)?

- Yes
- No 🡪(please continue with question 8)

Workdays

The following questions apply to the days on which you do your main work activities.

1. Please keep an average **workday** in mind, do you mainly **work at home**?

- Yes 🡪( please continue with question 8)
- No

1. **How many** days per week **do you commute to work**?

(for either a paid or an unpaid voluntary position)

- **1** day per week
- **2** days per week
- **3** days per week
- **4** days per week
- **5** days per week
- **6** days per week
- **7** days per week

1. Please keep an ordinary **workday** in mind, how many **hours per day**, do you commute using the following travel modes?

(please indicate what is applicable to your situation, multiple answers are allowed, please estimate durations)

| *Transport mode* | *autumn / winter* | *spring / summer* |
| --- | --- | --- |
| 🔾Train and Bus (Public transport) | hours minutes  _└─┴_ _─┘_ _└─┴─┘_ | hours minutes  _└─┴─┘_ _└─┴─┘_ |
| 🔾Car | hours minutes  _└─┴─┘_ _└─┴─┘_ | hours minutes  _└─┴─┘_ _└─┴─┘_ |
| 🔾Moped, scooter, motorbike | hours minutes  _└─┴─┘_ _└─┴─┘_ | hours minutes  _└─┴─┘_ _└─┴─┘_ |
| 🔾E-bike | hours minutes  _└─┴─┘_ _└─┴─┘_ | hours minutes  _└─┴─┘_ _└─┴─┘_ |
| 🔾Bicycle | hours minutes  _└─┴─┘_ _└─┴─┘_ | hours minutes  _└─┴─┘_ _└─┴─┘_ |
| 🔾On foot | hours minutes  _└─┴─┘_ _└─┴─┘_ | hours minutes  _└─┴─┘_ _└─┴─┘_ |
| 🔾Other transport mode,  (Namely): | hours minutes  _└─┴─┘_ _└─┴─┘_ | hours minutes  _└─┴─┘_ _└─┴─┘_ |

1. Do you have an “outdoors” occupation?

(your work activities are **mainly situated outdoors**, you are **multiple hours per day** outdoors carrying out your work activities)

- No
- Yes, I am _└────┘_ **hours per day** outdoors to do my work

1. Please keep an ordinary **workday** in mind, how many **hours per day**, do you spend traveling for work purposes, using the following travel modes?

(please indicate what is applicable to your situation, multiple answers are allowed, please estimate durations)

| *Transport mode* | *autumn / winter* | *spring / summer* |
| --- | --- | --- |
| 🔾None | _n.a_. | _n.a_. |
| 🔾Train and Bus (Public transport) | hours minutes  _└─┴─┘_ _└─┴─┘_ | hours minutes  _└─┴─┘_ _└─┴─┘_ |
| 🔾Car | hours minutes  _└─┴─┘_ _└─┴─┘_ | hours minutes  _└─┴─┘_ _└─┴─┘_ |
| 🔾Moped, scooter, motorbike | hours minutes  _└─┴─┘_ _└─┴─┘_ | hours minutes  _└─┴─┘_ _└─┴─┘_ |
| 🔾E-bike | hours minutes  _└─┴─┘_ _└─┴─┘_ | hours minutes  _└─┴─┘_ _└─┴─┘_ |
| 🔾Bicycle | hours minutes  _└─┴─┘_ _└─┴─┘_ | hours minutes  _└─┴─┘_ _└─┴─┘_ |
| 🔾On foot | hours minutes  _└─┴─┘_ _└─┴─┘_ | hours minutes  _└─┴─┘_ _└─┴─┘_ |
| 🔾Other transport mode,  (Namely): | hours minutes  _└─┴─┘_ _└─┴─┘_ | hours minutes  _└─┴─┘_ _└─┴─┘_ |

Leisure time

The following questions apply to periods when you are **not working**, or commuting to work, for instance during the weekends or at night.

1. Which of the following **outdoor leisure time activities** are in your **normal week schedule**?

(please indicate what is applicable to your situation, multiple answers are allowed, please estimate durations)

| *Activity* | *autumn / winter* | *spring / summer* |
| --- | --- | --- |
| 🔾Walking (e.g. while shopping, hiking, walking the dog) | Hours per week  _└─┴─┘_ | Hours per week  _└─┴─┘_ |
| 🔾Bicycle riding (e.g. from and to shops, bicycle tours) | Hours per week  _└─┴─┘_ | Hours per week  _└─┴─┘_ |
| 🔾Outdoor sports (e.g. running, tennis, football) | Hours per week  _└─┴─┘_ | Hours per week  _└─┴─┘_ |
| 🔾Spending time close to home (e.g. Time spent outdoors close to home, taking care  of animals, do-it-yourself work, relaxing in the garden) | Hours per week  _└─┴─┘_ | Hours per week  _└─┴─┘_ |
| 🔾Other outdoors activities (e.g. visiting a playground,  angling) | Hours per week  _└─┴─┘_ | Hours per week  _└─┴─┘_ |

1. How often do you use the following **transport modes per week during leisure time** and what are the **average durations per week** you use them?

(please indicate what is applicable to your situation, multiple answers are allowed, please estimate durations)

| *Transport mode* | *autumn / winter* | *spring / summer* |
| --- | --- | --- |
| 🔾Train and Bus (Public transport) | hours minutes  _└─┴─┘_ _└─┴─┘_ | hours minutes  _└─┴─┘_ _└─┴─┘_ |
| 🔾Car | hours minutes  _└─┴─┘_ _└─┴─┘_ | hours minutes  _└─┴─┘_ _└─┴─┘_ |
| 🔾Moped, scooter, motorbike | hours minutes  _└─┴─┘_ _└─┴─┘_ | hours minutes  _└─┴─┘_ _└─┴─┘_ |
| 🔾E-bike | hours minutes  _└─┴─┘_ _└─┴─┘_ | hours minutes  _└─┴─┘_ _└─┴─┘_ |
| 🔾Bicycle | hours minutes  _└─┴─┘_ _└─┴─┘_ | hours minutes  _└─┴─┘_ _└─┴─┘_ |
| 🔾On foot | hours minutes  _└─┴─┘_ _└─┴─┘_ | hours minutes  _└─┴─┘_ _└─┴─┘_ |
| 🔾Other transport mode,  (Namely): | hours minutes  _└─┴─┘_ _└─┴─┘_ | hours minutes  _└─┴─┘_ _└─┴─┘_ |

Closure

10.Please indicate below if you have any other remarks.

|  |
| --- |

***12.*** *Items from VGO study questionnaire (VGO questionnaire) (22,23, add. 3, add. 4)*

The answers to these questions were used as explanatory variables in the multiple linear regression analyses.

**A.2**  Please indicate your gender

0 Male

0 Female

**A.3** Please indicate your date of birth

└───┘ └───┘ └────────┘

Day Month Year

**A.4** What is your birth country?

0 the Nederlands

0 Another country, namely……………………

**B.4** Have you ever had asthma?

0 Yes 0 No

**B.5**  Was your asthma confirmed by a doctor?

0 No

0 Yes, it was confirmed in - - - - (year)

**B.12** Are you sensitive or allergic to the following substances?

A. House dust

B. Food items

C. Animals

D. Plants or pollen

E. Other substances, namely…………….

Question B.21 was a table indicating a range of health complaints: exhaustion, gastrointestinal complaints, nausea, diarrhoea, congestion, bloody/slimy excrements, being sick, fever, eye irritation, ear complaints, palpitations, neck or shoulder complaints, back complaints, chest pain, hand/wrist/elbow/arm complaints, leg/hip/knee/foot complaints, myalgia, headache, dizziness, anxious/nervous/tense feeling, feeling depressed, sudden stress or crisis, irritable/angry mood, sleeping problems, increased usage of alcohol/cigarettes/drugs/prescribed drugs, distress/shortness of breath while resting (without additional physical activity), sore throat, coughing, nasal complaints(e.g. often sneezing, irritated or stuffy nose, skin problems (itches, rash, red areas), urinary problems, changes in body weight. If any of these complaints were reported, follow-up question B.22 was also filled in.(55)

**B.22** Do you think that the health complaints you indicated, are possibly linked to the presence of livestock farms in the vicinity of your home?

0 Yes 0 No (if no, please continue with part C of the questionnaire)

**C.1** What is the highest level of education you completed? (add. 4)

0 None, did not complete any education

0 Primary school

0 Lower pre-vocational secondary school (LTS, LEAO, LHNO, VMBO)

0 Medium pre-vocational secondary school (MAVO, MULO, MBO-2/3yrs,

VMBO-t)

0 Senior secondary vocational education and training (MBO-4yrs, MTS,

MEAO, BOL, BBS, INAS)

0 Senior secondary education / university preparatory education (HAVO,

VWO, Atheneum, Gymnasium, HBS, MMS)

0 University of professional education (HBO, HTS, HEAO)

0 University

**D.2** Did you live on a livestock farm during your childhood (until age 18yrs)?

0 No

0 Yes, from….(years of age), until…..(years of age)

**D.15** Which pets did you keep during the past 5 years?

No, not in the past Yes, I currently Yes, I kept it during the last

5 years keep this pet 5 years, but not currently

Cat 0 0 0

Dog 0 0 0

Bird 0 0 0

Rabbit, hamster, 0 0 0

Guinea pig

Mouse or rat 0 0 0

Fish 0 0 0

Turtle 0 0 0

**D.17** Which hobby farm animals did you keep during the past 5 years?

No, not in the past Yes, I currently Yes, I kept it during the last

5 years keep this animal 5 years, but not currently

Pig 0 0 0

Cow 0 0 0

Sheep 0 0 0

Goat 0 0 0

Chicken, turkey, 0 0 0

duck, goose

Horse, pony, donkey 0 0 0

**E.1** Did you (ever) smoke cigarettes, cigars, and/or pipe tobacco? (yes, indicates at

least 20 packages in total or 1 year of at least 1 cigarette per day)

0 No

0 Yes, used to smoke, but quit …..years ago.

0Yes, I currently smoke

**References**

Add. 1 Quanjer PH, Stanojevic S, Cole TJ, Baur X, Hall GL, Culver BH, et al. Multi-

ethnic reference values for spirometry for the 3–95-yr age range: the global lung

function 2012 equations. Eur Respir J. 2012;40(6):1324–43.

Add. 2 The European Respiratory Health Survey. Respiratory Health Survey II. 2007;1–

65. http://www.ecrhs.org/Quests/ECRHSIImainquestionnaire.pdf.

Add. 3 Yzermans J, Baliatsas C, van Dulmen S, Van Kamp I. Assessing non-specific

symptoms in epidemiological studies: development and validation of the

symptoms and perceptions (SaP) questionnaire. Int J Hyg Environ Health

[Internet]. 2016;219(1):53–65. doi:10.1016/j.ijheh.2015.08.006.

Add. 4 NCEE [Internet]. http://www.ncee.org.
